# Gut microbiome and microbial metabolites in NAFLD and after bariatric surgery: Correlation and causality

**DOI:** 10.3389/fmicb.2022.1003755

**Published:** 2022-09-20

**Authors:** Yi Xia, Mengting Ren, Jinpu Yang, Changzhou Cai, Weixin Cheng, Xinxin Zhou, Dan Lu, Feng Ji

**Affiliations:** ^1^Department of Gastroenterology, The First Affiliated Hospital, Zhejiang University School of Medicine, Hangzhou, China; ^2^Department of Endoscopy Center, The First Affiliated Hospital, Zhejiang University School of Medicine, Hangzhou, China

**Keywords:** bariatric surgery, non-alcoholic fatty liver disease, metabolites, gut microbiome, gut-liver axis

## Abstract

Non-alcoholic fatty liver disease (NAFLD) is currently related to a heavy socioeconomic burden and increased incidence. Since obesity is the most prevalent risk factor for NAFLD, weight loss is an effective therapeutic solution. Bariatric surgery (BS), which can achieve long-term weight loss, improves the overall health of patients with NAFLD. The two most common surgeries are the Roux-en-Y gastric bypass and sleeve gastrectomy. The gut-liver axis is the complex network of cross-talking between the gut, its microbiome, and the liver. The gut microbiome, involved in the homeostasis of the gut-liver axis, is believed to play a significant role in the pathogenesis of NAFLD and the metabolic improvement after BS. Alterations in the gut microbiome in NAFLD have been confirmed compared to that in healthy individuals. The mechanisms linking the gut microbiome to NAFLD have been proposed, including increased intestinal permeability, higher energy intake, and other pathophysiological alterations. Interestingly, several correlation studies suggested that the gut microbial signatures after BS become more similar to those of lean, healthy controls than that of patients with NAFLD. The resolution of NAFLD after BS is related to changes in the gut microbiome and its metabolites. However, confirming a causal link remains challenging. This review summarizes characteristics of the gut microbiome in patients with NAFLD before and after BS and accumulates existing evidence about the underlying mechanisms of the gut microbiome.

## Introduction

Non-alcoholic fatty liver disease (NAFLD) includes a spectrum of liver conditions ranging from hepatic steatosis to non-alcoholic steatohepatitis (NASH), with or without fibrosis, that can develop into liver cirrhosis or hepatocellular carcinoma (Byrne and Targher, 2016). NAFLD is diagnosed by imaging or histological evidence of hepatic steatosis and the lack of secondary causes, such as excessive alcohol consumption, hepatitis, or hereditary disorders (Chalasani et al., 2018). Approximately 25% of the global adult population is affected by NAFLD, which confers a heavy socioeconomic pressure, comprising a predicted annual cost of $292 billion in the United States (Younossi et al., 2016; Huang et al., 2021). A relationship between NAFLD and metabolic comorbidities, including obesity and diabetes mellitus, has been established; additionally, obesity is the most documented and prevalent risk factor for NAFLD (Loomba et al., 2021). Therefore, the NAFLD population is predicted to increase by 18% by 2030 in correspondence with the increasing incidence of obesity and type 2 diabetes (Estes et al., 2018; Younossi et al., 2018).

Studies suggest that weight loss and healthy lifestyles can reverse NAFLD at an early stage (Romero-Gómez et al., 2017; Younossi et al., 2021). Bariatric surgery (BS) is the most efficient strategy for achieving long-term weight loss that also improves the overall health of patients with NAFLD (Cotter and Rinella, 2020; Syn et al., 2021). A systematic review and meta-analysis of data from 32 cohort studies comprising 3,093 biopsy specimens found that BS resulted in the biopsy-confirmed resolution of steatosis and fibrosis in 66 and 40% of patients with NAFLD, respectively. Furthermore, mean NAFLD activity scores are considerably reduced in patients after BS (Lee et al., 2019a).

However, the complex mechanisms of NAFLD improvement by BS remain to be clarified. Previous studies have elucidated the critical role of the gut microbiome (GM) in the homeostasis of the gut-liver axis and the pathogenesis of NAFLD (Tarao et al., 1977; Triger et al., 1978; Albillos et al., 2020). The gut microbial dysbiosis might contribute to NAFLD through several mechanisms, including increased energy harvesting and altered microbial production (Krawczyk et al., 2010). Indeed, some microbial changes in NAFLD, such as low microbial diversity and richness, can be reversed by BS. Also, altered microbial signatures are associated with metabolic improvements in NAFLD patients undergoing BS. Therefore, BS improves gut microbial dysbiosis, which may participate in NAFLD alleviation (Sun et al., 2019; Cerreto et al., 2021).

**FIGURE 1:**
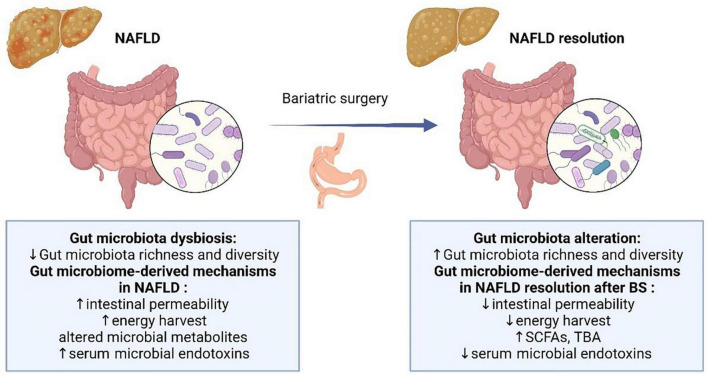
Overview of the gut microbial alteration and microbiome-derived mechanisms in NAFLD and after BS. NAFLD, non-alcoholic fatty liver disease; SCFA, short-chain fatty acids; TBA, total bile acids.

Here, we discuss the relationship between NAFLD and the gut microbiota and summarize recent advances in correlative and causality studies focusing on the role of the gut microbiome in NAFLD resolution after BS (Figure 1).

## Non-alcoholic fatty liver disease and gut microbiome

### The gut microbiome

The GM is a complex ecosystem consisting of bacteria, archaea, protists, fungi, and viruses living in the human gut (Martinez et al., 2016; Sender et al., 2016). The GM contains at least 40 trillion microorganisms comprising >1,000 species, approximately 90% of which are Bacteroidetes and Firmicutes, followed by Proteobacteria and Actinobacteria (Eckburg et al., 2005). Moreover, the gut microbiome plays an important role in host metabolism and homeostasis, including the prevention of pathogen colonization and coordination with immune reactions (Guarner and Malagelada, 2003). In addition, through a massive contribution to the pool of metabolites in the human systemic circulation, microbial metabolites systemically influence host metabolism and inflammation (Leung et al., 2016; Kolodziejczyk et al., 2019; Wu et al., 2021). Gut dysbiosis, which refers to the perturbation of the healthy gut microbiota, has been identified as a potential contributor to the development of many metabolite diseases, such as NAFLD, obesity, and diabetes (Qin et al., 2012; Liu et al., 2017). Recently, the development of methods to determine microbiome composition, such as the 16S ribosomal RNA (16S rRNA) sequencing and whole-genome shotgun sequencing, and functional analysis to determine microbial metabolic capacity have provided significant insight into the associations between the gut microbiota and related diseases (Hoozemans et al., 2021).

### Gut microbial signatures in non-alcoholic fatty liver disease

The gut microbial alterations in NAFLD patients have been observed in many clinical studies (Table 1). Gut microbial profiles differ, and the species richness notably decreases in patients with NAFLD. Compared with healthy persons, gram-negative (G−) bacteria are significantly enriched, while gram-positive (G+) bacteria are decreased in patients with NAFLD (Wang et al., 2016; Loomba et al., 2017). Specifically, *Bacteroidetes* and *Proteobacteria* are increased, whereas *Firmicutes* are decreased at the phylum level in patients with NAFLD (Zhu et al., 2013). Therefore, the ratio of *Firmicutes* to *Bacteroidetes* is an important factor in differentiating the gut microbiome of patients with NAFLD from that of healthy persons. However, trends can differ even among different families and genera in the same phylum. If only the phylum-level data are considered, changes in genera and families would be obscured, which is a disadvantage for distinguishing disease states based on higher phylogenetic levels (Raman et al., 2013). A decrease in the level of *Lachnospiraceae, Ruminococcaceae*, and *Veillonellaceae* families can explain the obvious depletion of the *Firmicutes* phylum in patients with NAFLD. The genus *Lactobacillus* (family *Lactobacillaceae*) is noticeably increased. The increased abundance of *Proteobacteria* in patients with NAFLD is mainly explained by that of the genus *Escherichia* (family *Enterobacteriaceae*) (Raman et al., 2013; Zhu et al., 2013).

**TABLE 1:** Clinical studies of the gut microbial changes in NAFLD patients.

Study	Groups	Microbiome	Metabolites
Rau et al., 2018	Healthy control (*n* = 27); NAFL (*n* = 14); NASH (*n* = 18)	NASH (compared to NAFL): α-diversity↓; *Fusobacteria↑; Fusobacteriaceae↑; Fusobacterium↑; Prevotella, ↑; Eubacterium biforme↑* NASH (compared to HC): *Fusobacteriaceae↑; Prevotellaceae↑* NAFL (compared to control): *Prevotellaceae↑*	NAFL and NASH (compared to control): Stool: propionate↑, butyrate↑, acetate↑
Kim et al., 2019	Non-NAFLD (G0) (*n* = 453); Developed NAFLD (G1) (*n* = 40); Regressed NAFLD (G2) (*n* = 35); Persistent (G3) (*n* = 238)	G3 (compared to G0): β-diversity↓, *Christensenellaceae*↓, *Odoribacteraceae*↓, *Oscillospira*↓, *Odoribacter*↓, *Coprococcus*↓, *Ruminococcaceae*↓, *Porphyromonadaceae*↓, *Christensenellaceae*↓, *Oscillospira*↓, *Ruminococcus*↓, *Coprococcus*↓	Not described
Caussy et al., 2019	Non-NAFLD control (*n* = 54); NAFLD without advanced fibrosis (*n* = 18); NAFLD-cirrhosis (*n* = 26)	NAFLD-cirrhosis (compared to control): *Streptococcus↑, Megasphaera↑, Bacillus*↓, *Lactococcus*↓,*Gallibacterium↑, Faecalibacterium prausnitzii↑, Catenibacterium↑, Rikenellaceae↑, Mogibacterium↑, Peptostreptococcaceae↑*	Not described
Chen F. et al., 2020	Lean control (*n* = 30); non-lean control (*n* = 46); Lean NAFLD (*n* = 99)	Lean NAFLD (compared to control): *Dorea↑, Marvinbryantia*↓, *Christensenellaceae R7*↓	Lean NAFLD (compared to control): higher total BA↑, total primary BA↑, total secondary BA↑, CDCA↑, DCA↑
Adams et al., 2020	Control (*n* = 55); NAFLD Fibrosis F0-2 (*n* = 58); NAFLD Fibrosis F3/4 (*n* = 9)	Increased NAFLD severity: α-diversity↓ *Firmicutes↑, Proteobacteria↑, Actinobacteria↑; Bacteroidetes*↓, *Actinomycetaceae↑, Lachnospiraceae↑, Bacteroidaceae*↓	Increased NAFLD severity: serum: total BA↑, primary conjugated BA↑, GCA↑, secondary conjugated BA↑; stool: total BA↑, DCA↑
Behary et al., 2021	Non-NAFLD control (*n* = 30); NAFLD-fibrosis (*n* = 28); NAFLD-HCC (*n* = 32)	NAFLD-HCC (compared to non-NAFLD): *Proteobacteria↑, Enterobacteriaceae↑, Oscillospiraceae*↓, *Erysipelotrichaceae*↓, NAFLD-cirrhosis (compared to non-NAFLD): *Eubacteriaceae↑, Coriobacteriaceae*↓, *Muribaculaceae*↓, *Odoribacteraceae*↓, *Prevotellaceae*↓ NAFLD-HCC (compared to NAFLD-cirrhosis): *Bacteroides caecimuris↑, Veillonella parvula↑*	NAFLD-HCC (compared to non-NAFLD): Stool: oxaloacetate↑, acetylphosphate↑, isocitrate↑, acetate↑, butyrate↑, formate↑ Serum: butyrate↑, propionate↑
Demir et al., 2022	Controls (*n* = 16); NAFL (*n* = 24); NASH (*n* = 54)	Non-obese F2-4 fibrosis (compared to non-obese F0-1): *Mucor sp.↑, Cyberlindnera jadinii↑, C. albicans↑, Salinispora sp.↑, Babjeviella inositovora↑*	Not described

SS, simple steatosis; NASH, non-alcoholic steatohepatitis; NAFLD, non-alcoholic fatty liver disease; HCC, hepatocellular carcinoma; HC, healthy control; BA, bile acid; CDCA, chenodeoxycholic acid; DCA, deoxycholic.

Changes in the gut microbiome composition of adults and children with NAFLD are similar. A comparison of the gut microbiomes between children with and without NASH found that NASH is associated with specific enterotypes of the gut microbiome. Most healthy gut microbiomes are classified as enterotypes 1 (enriched in *Bacteroides*) and 3 (diminished in *Bacteroides* and *Prevotella*), whereas obese and NASH gut microbiomes are more frequently classified as enterotype 2 (enriched in *Prevotella*) (Zhu et al., 2013). The *Gamma-* and *Epsilon-proteobacteria* at the phylum level and *Prevotella* at the genus level are more abundant in children with NAFLD than in healthy children. *Prevotella* is a typical genus in children with NAFLD (Michail et al., 2014).

Furthermore, gut microbiomes differ among patients with different NAFLD manifestations. The gut microbiome of patients with NAFLD becomes less diverse as NAFLD progresses with worsening fibrosis. A study of the association between gut dysbiosis and NAFLD severity found that *Bacteroides* and *Ruminococcus* are more prevalent, while *Prevotella* is less abundant in patients with *F* ≥ 2 fibrosis. Among these bacteria, the abundance of *Ruminococcus* and *Bacteroides* were independently associated with significant liver fibrosis (≥F2) and NASH, respectively (Boursier et al., 2016). A comparison of the gut microbiomes of patients with different stages of NAFLD found that the abundance of 37 species, including *Ruminococcus CAG: 39* and *Bacteroides caccae*, differed among mild, moderate, and advanced stages of NAFLD. A random forest classifier model has been constructed by identifying 40 features, including 37 bacterial species, to detect advanced fibrosis in NAFLD with diagnostic accuracy (Loomba et al., 2017). Moreover, the gut microbiome changes in NAFLD-related hepatocellular carcinoma (HCC), which might participate in NAFLD progression to HCC. The expansion of *Enterobacteriaceae* and *Eubacteriaceae* characterizes NAFLD-HCC and NAFLD-cirrhosis, respectively (Behary et al., 2021).

Interestingly, lean patients with NAFLD have different gut microbiota signatures and a more favorable metabolic profile than obese patients with NAFLD. Compared to obese patients with NAFLD, a marked deficiency in *Ruminococcus* and *Lactobacillus* genera and increase in the *Clostridium* genus and *Ruminococcaceae* family were found in lean patients with NASH (Duarte et al., 2018; Yun et al., 2019). The abundance of *Ruminococcus* might explain the smaller proportion of patients with liver fibrosis among lean patients with NAFLD. The *Ruminococcaceae* and *Clostridium* genera are involved in the formation of bile acids (BAs), which mediate resistance to diet-induced obesity (Watanabe et al., 2011; Chen F. et al., 2020). Compared with healthy controls, lean patients with NAFLD have an increased abundance of *Dorea*, suggesting NASH progression, and a decreased abundance of several species that protect against NAFLD, such as *Marvinbryantia* and *Christensenellaceae R7* group (Del Chierico et al., 2016; Zhou et al., 2017). Although enriched pathogenic bacteria in the gut microbiome play an important role in the increased susceptibility of non-obese persons at high risk for NAFLD, their gut microbiomes also change to maintain homeostatic responses. As NAFLD develops, interactions among complex systemic processes lead to the failure of metabolic adaptation in lean patients with NAFLD (Chen F. et al., 2020).

Consistent microbiota features have not been identified in NAFLD because of controversial microbiome profiles among studies. Such inconsistency might be due to factors, including different methodology, characteristics of NAFLD patients, such as ethnicity, diet, environment, disease stages, and associated comorbidities, such as diabetes and other metabolic syndromes, and so on (Raman et al., 2013). Therefore, the microbial signatures in NAFLD require further homogeneous and large-scale investigation.

## Microbiome-derived mechanisms in non-alcoholic fatty liver disease

**FIGURE 2:**
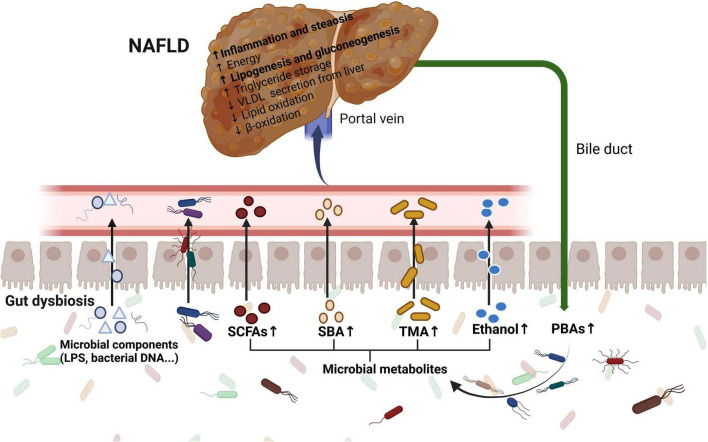
The role of the gut microbiome in the pathogenesis of NAFLD. Gut dysbiosis leads to increased intestinal permeability, increased dietary energy harvest, altered microbial metabolites including SCFAs, BAs, TMA, and ethanol, and increased microbial endotoxins. The communication *via* systemic mediators is not shown. LPS, lipopolysaccharide; SCFAs, short-chain fatty acids; SBAs, secondary bile acids; PBAs, primary bile acids; TMA, trimethylamine; VLDL, very low-density lipoprotein.

The underlying mechanisms of the gut microbiome and related metabolites in NAFLD have been investigated in recent years. Suggested mechanisms include increased intestinal permeability, increased dietary energy harvest, altered microbial metabolites, such as short-chain fatty acids (SCFAs) and BAs, and increased microbial endotoxins (Figure 2).

### Increased intestinal permeability

The intestinal barrier, as part of the gut-liver axis, consists of the structural elements including tight junctional complexes and mucus layer, immune cells, and soluble mediators such as antimicrobial peptides (De Munck et al., 2020). A healthy intestinal barrier separates the host from the gut contents (Kolodziejczyk et al., 2019). An imbalance in the gut microbiome composition and function leads to a disrupted intestinal barrier, which increases intestinal permeability (Safari and Gérard, 2019). Enhanced intestinal permeability leads to the translocation of bacteria and their products into the portal circulation, and increases hepatic exposure to injurious substances that might subsequently cause the development of NAFLD (Turner, 2009). A meta-analysis based on 14 studies showed that intestinal permeability increased in NAFLD patients compared to healthy controls and was associated with the degree of hepatic steatosis (De Munck et al., 2020). Also, the decreased expressions of major tight junction proteins, zonula occludens-1 (ZO-1), and junctional adhesion molecule A (JAM-A) have been found in the intestinal mucosa of NAFLD patients (Miele et al., 2009; Rahman et al., 2016).

The role of the gut microbiome on the mucus layer and the epithelial and vascular barriers has been studied in mice and humans. Mice fed with a high-fat diet (HFD) are protected from increased intestinal permeability in the absence of the gut microbiota (Thaiss et al., 2018). The permeability of the small intestine of patients with NAFLD is decreased after receiving an allogenic fecal microbiota transplant (FMT) from lean, healthy donors (Craven et al., 2020). This revealed a relationship between gut dysbiosis and intestinal permeability. Gut microbiome dysbiosis degrades mucus or inhibits its production, thus altering the mucus layer (Groschwitz and Hogan, 2009).

While the relationship between the gut microbiome and intestinal permeability has been verified, the relationship between gut barrier dysfunction and NAFLD remains unclear. A diet-induced (methionine-and-choline-deficient; MCD) murine model of NASH showed liver injury in the early stage before any change in intestinal permeability. This suggested that the initial liver damage phase might be contributing to the observed intestinal permeability (Luther et al., 2015). However, JAM-A-deficient mice with defects in intestinal epithelial permeability developed more severe steatohepatitis than control mice after a high-fat, fructose, and cholesterol diet (Rahman et al., 2016). Therefore, further investigation is required to confirm the mechanisms underlying gut permeability and NAFLD.

### Increased energy harvest

The human gut microbiome contributes to the processing of dietary components, such as fats and carbohydrates that influence energy harvesting and lead to metabolic syndromes. Patients with NAFLD have more pathways of energy production and conversion, which indicate increased energy and caloric retention.

Altered gut microbiomes play key regulatory roles in energy extraction and are closely associated with fat deposition in the livers of patients. The gut microbiome is enriched in six functional categories associated with carbohydrate metabolism, lipid synthesis, amino acids metabolism, and secondary metabolism in patients with NASH (Boursier et al., 2016). Pathways associated with energy production and conversion are notably more abundant in obese than in lean patients with NAFLD, which might explain why obese children with NAFLD gain weight even when their dietary intake is similar to that of healthy children (Michail et al., 2014). Furthermore, total body fat significantly increases in germ-free (GF) mice transplanted with gut microbiome from obese mice as compared with that from lean mice with similar food intake (Turnbaugh et al., 2006).

### Altered microbial metabolites

Gut microbial metabolites function as bacterial messengers in the gut microbiota and host metabolism. Several metabolites contribute to NAFLD pathogenesis in animals.

#### Short-chain fatty acids

Short-chain fatty acids, including acetate, butyrate, and propionate, fermented from indigestible dietary fibers by intestinal bacteria, are among the most abundant microbial metabolites in the gut. *Bacteroides*, *Anaerostipes*, and other gut bacteria are primarily involved in the production of SCFAs that play important roles in gut integrity, lipid metabolism, glucose homeostasis, appetite regulation, and immune responses (Morrison and Preston, 2016). The fecal concentration of SCFAs is higher and SCFA-producing bacteria are more abundant in patients with NAFLD than in healthy controls. Fecal propionate levels gradually increase with the increasing severity of fibrosis in patients with NAFLD (Lee G. et al., 2020). A study of patients and healthy controls matched for body mass index (BMI) to exclude the influence of obesity on SCFA found higher concentrations of fecal acetate, butyrate, and formate, as well as serum butyrate and propionate in patients with NAFLD-HCC than in patients with NAFLD-cirrhosis and healthy controls (Behary et al., 2021).

Short-chain fatty acids are associated with NAFLD *via* several mechanisms. They increase insulin sensitivity and reduce hepatic fat storage. Their effects are exerted mainly through activation of the G protein-coupled receptors (GPCRs), GPR41, and GPR43 that induce peptide YY (PYY) release and increase the secretion of glucagon-like peptide 1 (GLP-1) (Samuel et al., 2008; Bellahcene et al., 2013). Both PYY and GLP-1 impede gastric emptying and increase satiety (Svegliati-Baroni et al., 2011). SCFAs also inhibit chylomicron secretion, promote lipid oxidation, and alleviate insulin resistance *via* an adenosine monophosphate-activated protein kinase (AMPK)-dependent mechanism. Moreover, SCFAs help to maintain the gut barrier permeability and decrease lipopolysaccharide (LPS) concentrations in the portal vein by improving transepithelial electrical resistance (Zhao et al., 2018). They also inhibit liver inflammation by negatively regulating NF-κβ and decreasing the secretion of pro-inflammatory factors, such as TNF-α and IL-1β (Park et al., 2019). However, excessive SCFAs may inhibit AMPK in the liver and increase the accumulation of hepatic free fatty acids (FFA) *via β*-oxidation (Leung et al., 2016). SCFAs also induce pro-inflammatory T cells, such as Th1 and TH17, under specific conditions. Fecal propionate and acetate concentrations significantly and positively correlate with the peripheral Th17/resting-Treg (rTreg) ratio and negatively with peripheral rTregs that are the immunological features of a progressive disease (Rau et al., 2018).

Thus, the role of SCFAs in patients with NAFLD remains controversial. The contradictory results of preclinical and clinical studies warrant further investigation to identify the molecular mechanisms of SCFAs in NAFLD pathogenesis.

#### Bile acids

The liver synthesizes BAs from cholesterol as primary bile acids (PBAs), such as cholic acid (CA) and chenodeoxycholic acid (CDCA). These are deconjugated and dehydroxylated by the gut microbiota to secondary bile acids (SBAs), such as deoxycholic (DCA) and lithocholic (LCA) acids, that are reabsorbed in the distal ileum and returned to the liver through the portal vein (Santos et al., 2020). Specific gut microbes, such as *Bacteroidaceae* (order *Bacteroidales*) and *Lachnospiraceae* families, correlate with fecal BA concentrations (Adams et al., 2020). Bile acids play important roles in the pathogenesis and development of NAFLD and act as a bridge between the gut microbiome and liver.

As signaling molecules that regulate glucose, lipid, and inflammation, BAs contribute to hosting metabolism mainly *via* Farnesoid X receptor (FXR) and Takeda G-protein-coupled receptor 5 (TGR5). Upon activation, mainly, by PBAs, FXR induces fibroblast growth factor 19 (FGF19) to enhance glucose uptake in adipocytes by activating the mammalian target of rapamycin complex 1 (mTORC1) *via* mitogen-activated protein kinase (MAPK). However, FXR also inhibits the expression of sterol regulatory element-binding protein 1c (SREBP-1c) and induces FXR-dependent peroxisome proliferator-activated receptor alpha (PPARα) to limit hepatic lipid accumulation and increase fatty acid β-oxidation (Watanabe et al., 2004; Carr and Reid, 2015; Fuchs et al., 2016). An FXR agonist exerts antisteatotic effects in mice fed with HFD, which inhibits the expression of perilipin 2, a lipid droplet protein that is abundantly expressed in patients with NAFLD (Liu et al., 2014). In contrast, the TGR5 activated by SBAs induces the release of glucagon-like peptide-1 (GLP-1), which increases insulin synthesis and decreases appetite and food intake (Lund et al., 2020). In addition, the activation of FXR and TGR5 suppresses the expression of cholesterol 7α-hydroxylase (CYP7A1), which is the rate-limiting enzyme in synthesis, to achieve BA feedback regulation.

However, gut dysbiosis increases the PBA/SBA ratio and influences the functions of BAs *via* TGR5 and FXR in patients with NAFLD. Serum and fecal BA concentrations increase in patients with NAFLD. Increased serum BAs are driven by primary and secondary conjugated BAs, whereas fecal BAs are driven by secondary unconjugated BAs (Adams et al., 2020). During NAFLD development, total primary BAs increase and total secondary BAs decrease stepwise from healthy controls to NAFLD to NASH (Puri et al., 2017). Specifically, the proportions of serum glycocholic (GCA) and glycodeoxycholic (GDCA) acids in total PBAs and SBAs are respectively associated with advanced liver fibrosis in NAFLD. Furthermore, fecal DCA is associated with advanced fibrosis (Adams et al., 2020). The concentration of total serum SBAs containing GCA is notably higher in lean than in obese patients with NAFLD (Chen F. et al., 2020). Although BA levels are increased in patients with NAFLD, FXR-mediated signaling is inhibited, and the concentration of circulating FGF19 is decreased (Jiao et al., 2018; Nobili et al., 2018). The composition of BAs is significantly changed and weight gain and hepatic steatosis are promoted in FXR-deficient mice fed with an HFD (Parséus et al., 2017). The expression of CYP7B1 is upregulated in hamsters with an ablated gut microbiota fed with HFD, which leads to a more hydrophilic BA composition with an increased abundance of tauro-β-muricholic acid (TβMCA), an endogenous FXR antagonist. In addition, inhibited hepatic FXR signaling in hamsters treated with antibiotics is associated with increased TβMCA and reduced DCA and LCA. These findings highlighted microbial BA modulation as an underlying mechanism of obesity-induced metabolic disorders through influencing intestinal FXR (Sun et al., 2019).

#### Choline and trimethylamine-N-oxide

Choline is an important metabolite obtained *via* dietary intake and endogenous synthesis that is implicated in the pathogenesis of NAFLD and NASH. Choline is an essential cell membrane phospholipid required for hepatic low-density lipoprotein (VLDL) production (Corbin and Zeisel, 2012). Therefore, a choline deficiency might lead to decreased hepatic VLDL production along with triglyceride (TG) accumulation that further causes liver steatosis and NASH (Yao and Vance, 1990; Li et al., 2019).

The gut microbiome can convert choline to trimethylamine (TMA) and phosphatidylcholine. Trimethylamine is further oxidized by hepatic monooxygenase and metabolized to trimethylamine-N-oxide (TMAO) in the liver (Wang et al., 2011). The features of microbiota-driven choline metabolism in patients with NASH are increased conversion of choline to TMA and decreased choline bioavailability. The major bacterial phyla contributing to this are *Proteobacteria*, *Firmicutes*, and *Actinobacteria*. The increased concentrations of TMA and TMAO are associated with an elevated ratio of *Firmicutes* to *Bacteroidetes*, which is also a feature of the gut microbial composition in patients with NAFLD (Falony et al., 2015; Martínez-del Campo et al., 2015; Cho et al., 2017). Increased serum TMAO concentrations in patients with NAFLD compared with healthy controls are also related to the severity of NAFLD (Chen et al., 2016). A specific cut-off for TMAO might help to identify individuals at high risk for NAFLD who required specific nutritional intervention (Barrea et al., 2018). Although the mechanism of the TMAO contribution to NAFLD remains unclear, a high urinary excretion of TMAO is associated with insulin resistance in mice fed with HFD (Dumas et al., 2006). Administering TMAO to mouse models of HFD-induced NAFLD and aggravated liver steatosis by shifting the hepatic BA composition to FXR-antagonism and inhibiting the hepatic FXR signaling that consequently upregulates lipogenesis (Tan et al., 2019). Furthermore, TMAO increases serum cytokine C-C motif chemokine 2 (CCL2) levels and causes inflammation in adipose tissues (Gao et al., 2014).

#### Endogenous ethanol

Ethanol is a microbial metabolite found mainly in human blood. Its concentration is remarkably increased by consuming carbohydrate-rich diets (Sarkola and Eriksson, 2001). Patients with NAFLD harbor an increased abundance of alcohol-producing gut bacteria, such as *Escherichia, Gammaproteobacteria*, and *Prevotella*, compared with healthy controls (Ren et al., 2007; Jiang et al., 2015). Three major hepatic alcohol metabolizing pathways are also upregulated in patients with NAFLD (Baker et al., 2010; Zhu et al., 2016). These changes lead to an increased concentration of blood ethanol in patients with NASH who do not consume alcohol (Zhu et al., 2013; Aragonès et al., 2020). Blood levels of ethanol are significantly increased and positively associated with blood levels of insulin, leptin, and triglycerides in children with NASH (Engstler et al., 2016).

Ethanol is related to the pathogenesis and development of NASH and NAFLD. Ethanol metabolism stimulates lipogenesis *de novo* and decreases fatty acid (FA) oxidation, both of which result in liver steatosis (Lee et al., 2019b). Furthermore, ethanol increases gut permeability, causes endotoxemia by inducing inflammatory cytokine expression *via* the NF-κB pathway, and disrupts the apical junctional complexes in the colonic epithelium (Rao et al., 2004). Ethanol metabolism also produces reactive oxygen species (ROS) and aggravates oxidative stress by inducing the cytochrome P450 2E1 (CYP2E1) expression, which leads to liver injury (Lieber, 1999). Endogenous ethanol produced by bacteria impairs mitochondrial integrity and contributes to NAFLD development by inducing mitochondrial ROS production and mitochondrial DNA damage (Chen X. et al., 2020). Moreover, acetaldehyde produced from ethanol catalyzed by alcohol dehydrogenase (ADH), is converted to acetate by CYP2E1. When this pathway is saturated, acetaldehyde accumulates and causes hepatotoxicity (Miura, 2014).

In conclusion, high levels of endogenous ethanol are produced by the gut microbiome, as gut dysbiosis induces the pathogenesis and development of NAFLD by impairing the intestinal barrier, increasing toxicity in hepatic cells, and inducing inflammation. Further studies are needed to determine the underlying mechanisms of ethanol and confirm its functions as a therapeutic target for NAFLD.

### Microbial endotoxins

Blood concentrations of endotoxic lipopolysaccharides (LPS) released from the cell walls of G^–^ bacteria are low in healthy persons because the gut microbiome not only safeguards the gut barrier integrity but also has anti-inflammatory functions. However, an increased abundance of G^–^ bacteria, such as *Proteobacteria*, *Enterobacteria*, and *Escherichia*, with gut permeability leads to increased LPS levels in the gut and blood of patients with NAFLD. Plasma endotoxin levels and markers of inflammation are significantly higher in patients with NAFLD than in age-matched controls, which increase with the severity of hepatic steatosis (Nier et al., 2020). Non-virulent endotoxin-producing microbial species of pathogenic species overgrowing in the obese human gut cause the induction of NAFLD. Therefore, the overgrowth of these bacteria might collectively serve as a predictive biomarker of NAFLD (Fei et al., 2020).

The main mechanism of NASH is associated with LPS and other bacterial products, such as peptidoglycan, flagellin, and bacterial DNA, which are recognized by the Toll-like receptors (TLRs), including TLR2, TLR4, TLR5, and TLR9 (Kawai and Akira, 2009, 2010; Miura et al., 2010). Mice with NAFLD accompanied by a TLR4 deficiency have less liver injury, inflammation, and lipid accumulation than wild-type mice with NAFLD (Rivera et al., 2007). Ligands of TLR stimulate cells, such as macrophages, to express TLR by activating NF-κβ to produce pro-inflammatory cytokines including tumor necrosis factor α (TNFα) and interleukin-1β (IL-1β), which participate in lipid metabolism and induce hepatocyte cell death (Hotamisligil et al., 1994; Carpino et al., 2020). Macrophages also generate chemokines, such as monocyte chemoattractant protein-1 (MCP-1), to recruit inflammatory macrophages, which stimulate hepatic stellate cells and lead to liver fibrosis together with specific TLR ligands (Stienstra et al., 2010; Miura, 2014). In addition, a “leaky gut” leads to bacterial translocation, which is the migration of viable bacteria and their products from the intestinal lumen to the mesenteric lymph node complex (Festi et al., 2014). High concentrations of LPS and other bacterial metabolites cause endotoxemia and stimulate the transcription of inflammatory genes through the NF-κB pathway (Brun et al., 2007; Compare et al., 2016). Furthermore, LPS and other bacterial products can be detected by NOF-like receptor pyrin domain-containing 3 (NLRP3), which might form inflammasomes with other proteins and stimulate immunity (Yang et al., 2016). The NLRP3 selective inhibitor, MCC950 (also known as the cytokine release *inhibitory* drug 3; CRID3) improves NAFLD pathology and fibrosis in obese diabetic mice (Mridha et al., 2017). These mechanisms explain the effects of endotoxins on the pathogenesis of NAFLD.

## Bariatric surgery and non-alcoholic fatty liver disease

### Overview of bariatric surgery

Bariatric surgery is a proven, effective, and durable therapy for patients with BMI ≥ 40 kg/m^2^, BMI between 35 and 39.9 kg/m^2^, and poor glycemic control (Cummings and Rubino, 2018). Moreover, BS is classified into the following categories according to the applied procedures as:

1. Restrictive procedures to decrease stomach size and restrict food intake, such as vertical banded gastroplasty (VBG), gastric banding (GB), sleeve gastrectomy (SG), and gastric imbrication.
2. Malabsorptive procedures that short the small intestine to decrease the absorption of nutrients.
3. Biliopancreatic diversion (BPD), Roux-en-Y gastric bypass (RYGB), and single-anastomosis gastric bypass (SAGB) (Cerreto et al., 2021).

The RYGB and SG procedures are the most frequently applied types of BS worldwide and are addressed here (Nguyen and Varela, 2017). In the past years, SG has been the primary BS, and studies on SG compared to RYGB are still missing. The RYGB involves a gastric pouch creation that is anastomosed to the distal jejunum by the Roux limb. Restrictive and malabsorptive strategies are combined in the RYGB procedure to achieve weight loss. They include reduction of the stomach volume and the consequent slow the gastric pouch emptying, which leads to early satiation and decreased food and energy intake while bypassing the distal stomach, duodenum, and jejunum, to reduce the digestion and absorption of micro- and macronutrients (Cerreto et al., 2021). SG is a purely restrictive procedure in which approximately removes 80% of the stomach and the remainder of the stomach is fashioned into a narrow tube or sleeve to reduce food intake (Bult et al., 2008). Both RYGB and SG alter the secretion of the gut hormones. Decreased levels of ghrelin and increased levels of GLP-1 and PYY reduce hunger and increase satiation and satiety independently of weight loss for up to 10 years (Dar et al., 2012; Yousseif et al., 2014). Changes in the composition and circulating concentrations of BAs after RYGB and SG also contribute to postoperative metabolic effects (Steinert et al., 2013).

### Resolution of non-alcoholic fatty liver disease after bariatric surgery

Lifestyle modifications focused on weight loss remain the cornerstone of NAFLD management (Romero-Gómez et al., 2017). The most effective method to achieve long-term weight loss is BS (Cornejo-Pareja et al., 2021). In addition, BS modulates metabolic factors, such as glycemia, insulin sensitivity, and lipid metabolism (Hutch and Sandoval, 2017). Additionally, BS confers benefits on NAFLD by improving hepatic injury, hepatic fat, and the histological features of NAFLD, independently of weight loss. Clinical, biological, and histological data collected from 109 morbidly obese patients with biopsy-proven NASH revealed that the mean NAFLD scores were reduced from 5 to 1, and that mild and severe NASH disappeared in 94 and 70% of patients, respectively, a year after the surgery (Lassailly et al., 2015). Histological remission of NASH has been identified in liver samples from 84% of patients 5 years after BS; fibrosis becomes progressively reduced over 1 to 5 years (Lassailly et al., 2020). A retrospective analysis also independently associated BS with a decreased risk of developing cirrhosis in 2,942 patients with NAFLD (Wirth et al., 2020). The degree of weight loss after BS predicts the extent of improvement in NAFLD fibrosis scores (Yeo et al., 2019). The nature of BS procedures appears to have different effects on NAFLD. For example, liver stiffness is improved in patients after RYGB than laparoscopic sleeve gastrectomy (LSG) (Nickel et al., 2018). A systematic review and meta-analysis found that NAFLD resolution is more complete in proportion to RYGB across all liver histological features, including steatosis and inflammation, compared with combined analyses (Lee et al., 2019c). However, another meta-analysis found no differences in the histopathological outcomes of RYGB and SG in patients with NAFLD. Therefore, large-scale studies and more rigorous analyses are needed to confirm the effects of BS on NAFLD (de Brito et al., 2021).

The underlying mechanisms of NAFLD resolution by BS have been investigated. Obese rats on HFD underwent a duodenojejunal bypass (DJB) or sham operations, and were pair-fed for 15 weeks postoperatively to match their weight. The results proved that BS directly affected hepatic fat accumulation and insulin resistance independent of weight reduction (Angelini et al., 2020). Further investigation in rodents and humans has revealed that reduced caloric intake after SG increases the expression of phosphorylated AMPK, which is a crucial step in Plin2-LAMP2A binding; this leads to enhanced autophagy of Plin2 that exposes LD triglycerides to intracellular lipases (Angelini et al., 2019). Both SG and RYGB induce the downregulation of angiopoietin-like 8 (ANGPTL8), which inhibits lipogenesis in human hepatocytes when exposed to lipotoxic conditions and is associated with the degree of steatosis in the livers of rats with diet-induced obesity. These findings support the suppose that ANGPTL8 partly improves NAFLD after BS by improving hepatic lipid metabolism (Perdomo et al., 2021). Bile acid signaling also contributes to the resolution of NAFLD after BS, as RYGB causes an increase in total BAs, and this is related to the normalized accumulation of liver fat. Improvements in NAFLD after RYGB are attenuated by inhibiting PPARα (Mazzini et al., 2021). The complex mechanisms of BS in resolving NAFLD, including the gut microbiome and gastrointestinal hormones, require comprehensive investigation.

## Role of gut microbiome in non-alcoholic fatty liver disease resolution after bariatric surgery

**FIGURE 3:**
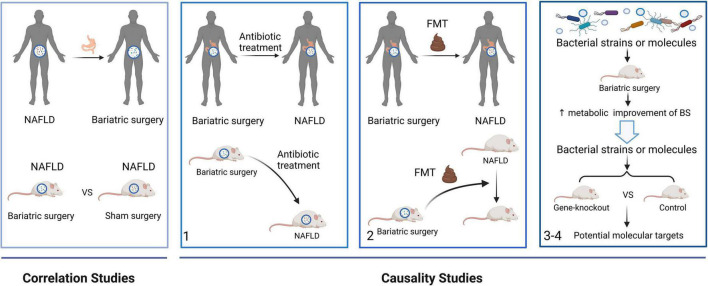
A framework to study the role of the gut microbiome on the resolution of NAFLD after BS from correlation to causation. Correlations studies revealed the gut microbial alterations after BS in NAFLD patients and mice. In causality studies, firstly, antibiotic treatment attenuates the metabolic improvement of BS in NAFLD. Secondly, the metabolic benefits of BS are transferred using FMTs. Finally, microbial strains or molecules enhanced the effects of BS. Moreover, gene-knockout mice are used to find the molecular mechanism of the gut microbiome. FMT, fecal microbiota transplant.

As environmental factors and anatomical structures change in the digestive tract, BS modifies obesity-related metabolomic fingerprints, especially those associated with the gut microbiota and microbial metabolites, and induces metabolic improvements by mimicking the metabolome and microbiome associated with a healthy gut (Liu et al., 2017; Sánchez-Alcoholado et al., 2019; Chaudhari et al., 2021a). Correlations between causal factors and microbiome-linked diseases have recently been revealed (Chaudhari et al., 2021b). Therefore, this section explores the influence of gut microbiome on the resolution of NAFLD after BS from the viewpoints of correlations and causality (Figure 3).

### Correlation studies

Table 2 selectively lists some clinical studies evaluating the gut microbial alterations after BS.

**TABLE 2:** Clinical studies evaluating the gut microbial alterations after bariatric surgery.

Study	Groups	Follow-up	Microbiome	Metabolites
Liu et al., 2017	SG (*n* = 23);	3 months	*C. comes↑, D. longicatena↑, Clostridiales↑, Anaerotruncus↑, colihominis↑, A. muciniphila↑*, *B. thetaiotaomicron↑*	AAA↓, methionine↓, alanine↓, lysine↓, serine↓, glutamate↓, acetyglycine↑, glycine↑; The increase in abundance of *B. thetaiotaomicron* after SG was associated with the decrease in circulating glutamate levels.
Ilhan et al., 2017	RYGB (*n* = 24); LAGB (*n* = 14)	/	RYGB vs. obesity patients: *Bacilli↑, Gammaproteobacteria↑, Prevotellaceae↑, Trabulsiella↑, Coprococcus↑, Oscillospira*↓, *Coprobacillus*↓, *Holdemania*↓, *Bacteroides*↓ RYGB vs. LAGB: Microbiota diversity↑, *Gammaproteobacteria↑, Bacilli↑Bacteroidaceae↑*, LAGB vs. obesity patients: *Flavobacteriia↑ Porphyromonadacea↑*	Increased abundance of butyrate, propionate and branched chain fatty acids only be found in RYGB group
de Jonge et al., 2019	the duodenal– jejunal bypass liner (DJBL) (*n* = 17)	6 months	6 months: *Lactobacillus gasseri↑, Lactobacillus plantarum↑, Veillonella spp.↑, Enterobacter aerogenes↑* *Escherichia coli↑, Serratia↑*	Not described
Sánchez-Alcoholado et al., 2019	RYGB (*n* = 14); LSG (*n* = 14)	3 months	LSG: *Lentisphaerae↑, Victivillaceae↑, Akkermansi↑, Eubacterium↑, Blautia↑, Haemophilus↑, Clostridiaceae*↓, *Anaerostipes*↓*Bififidobacterium*↓, *Bififidobacteriaceae*↓ RYGB: *Fusobacteriaceae↑, Clostridiaceae↑, Enterobacteriaceae↑, Proteobacteria↑Bififidobacteriaceae*↓ *Peptostreptococcaceae*↓, *Collinsella*↓, *Clostridium↑*, *Veillonella↑, Fusobacterium↑, Slackia↑, Granucatiella↑* *Oscillospira↑*	Not described
Fouladi et al., 2019	RYGB (*n* = 12)	3 days	post-RYGB patients who experienced successful weight loss: *Verrucomicrobiae↑, Bacilli↑, Lactobacillales↑Enterobacteriales↑, Senegalimassilia↑, Rothia↑* post-RYGB patients who experienced poor weight loss: *Bacilli↑, Lactobacillales↑, Enterobacteriales↑, Rothia↑* *Streptococcus↑*	post-RYGB: CA, CDCA, DCA, TBA↑
Lee et al., 2019a	AGB (*n* = 4); RYGB (*n* = 4); Medical weight loss (MWL, *n* = 4)	until 10% weight loss	RYGB: alpha diversity↑, *Proteobacteria↑, Actinobacteria↑Faecalibacterium↑;* AGB: *Proteobacteria↑;* MWL: alpha diversity↑	Not described
Mabey et al., 2020	RYGB (*n* = 16);	13 years	Post-RYGB: *Verrucomicrobiaceae↑, Streptococcaceae↑, Bacteroidaceae*↓	Not described
Li et al., 2021	RYGB (*n* = 68); SG (*n* = 10); LGB (*n* = 6)	12 months	Post RYGB: *Klebsiella↑, Escherichia_Shigella↑* *Streptococcus↑, Veillonella↑, Adlercreutzia↑, Alistipes↑, Barnesiella↑, Parabacteroides↑, Clostridium_XIVa↑, Coprococcus↑*	Post RYGB: Urinary excretion of 4-hydroxyphenylacetate, phenylacetylglutamine, 4-cresylsulfate, and indoxyl sulfate↑; Fecal excretion of tyramine and phenylacetate↑; circulating levels of dimethyl sulfone↑ and BCAAs↓
Fukuda et al., 2022	LSG (*n* = 10)	12 months	*Bacteroidetes↑, Fusobacteria↑*, alpha diversity↑	Not described
Scheithauer et al., 2022	RYGB (*n* = 40)	12 months	*Proteobacteria↑, Firmicutes*↓, alpha diversity↑ most anaerobic bacteria↑	Not described
Han et al., 2022	RYGB (*n* = 22), SG (*n* = 30)	12 months	Post-surgery: *Fusobacteria↑, Proteobacteria↑, Verrucomicrobia↑, Streptococcus↑, Oscillospirav↑, Akkermansia↑, Bifidobacterium*↓, *Turicibacter*↓, *Prevotella*↓	Post-surgery: BCAAs↓, AAAs↓, isoleucine↓, leucine↓, valine↓, tyrosine↓, alanine↓, glucose↓, glutamate↓, lactate↓, mannose↓, DMSO2↑, glycine metabolites↑, TBA↑, GDCA↑, TDCA↑, LCA↑, GLCA↑, TLCA↑

LAGB, laparoscopic adjustable gastric banding; RYGB, Roux-en-Y gastric bypass; LSG, laparoscopic sleeve gastrectomy; AAA, aromatic amino acids; BCAA, branched-chain amino acids; CA, cholic acid; DCA, deoxycholic acid; CDCA, chenodeoxycholic acid; TBA, total bile acid; GDCA, glycodeoxycholic acid; TDCA, tauroursodeoxycholic acid; LCA, lithocholic acid; GLCA, Glycocholic acid; TLCA, Taurolithocholic acid.

#### Gut microbial composition after bariatric surgery

Gut microbial signatures become similar to those of lean, healthy controls than that of patients with NAFLD. Some microbial changes, such as decreased *Lactobacillales* and increased *Dorea* as deleterious consequences of NAFLD, can be reversed after BS. Systematic reviews of clinical studies and animal experiments have found increased microbial diversity and richness after BS compared with that in patients with NAFLD. In addition, the abundance of *Bacteroidetes, Fusobacteria, Verrucomicrobia*, and *Proteobacteria* phyla increases after surgery. The abundance of *Lactobacillales, Enterococcus, Gammaproteobacteria, Akkermansia, and Enterobacteriales* phyla also increases; however, that of *Firmicutes, Clostridiales, Clostridiaceae, Blautia, and Dorea* phyla decreases (Guo et al., 2018). However, RYGB causes more alterations in the gut microbial composition than SG. Decreases in the abundance of *Firmicutes* is more significant after SG, whereas increase in the concentration of *Bacteroidetes* and *Proteobacteria* are more remarkable after RYGB (Davies et al., 2019). Because of the different changes in anatomical structures and gut environments between RYGB and SG, aero-tolerant bacteria, such as *Streptococcus* and *Veillonella spp*., become more abundant after RYGB, whereas anaerobes, such as *Clostridium*, become more abundant after SG (Farin et al., 2020). In addition, weight-loss-associated oral microbial phylotypes are increased in the fecal microbiome after RYGB, which might be due to decreased gastric acid exposure (Ilhan et al., 2017). Unlike energy restriction, BS leads to long-term effects on the gut microbiome, which result in the remission of metabolic diseases. A 12-year follow-up study of patients after RYGB uncovered a higher concentration of *Verrucomicrobiaceae* and *Streptococcaceae* and a lower abundance of *Bacteroidaceae* 10.6 years after RYGB as compared with controls.

Altered microbial signatures are associated with metabolic improvements in patients undergoing BS. Changes in the gut microbiome are related to host metabolic parameters after BS. *Blautia* and *Streptococcus* are positively and negatively associated with high-density lipid-cholesterol (HDL-C), respectively. *Bacteroides* correlates with heptanoate levels. *Bifidobacterium* correlates with total cholesterol, low-density lipoprotein cholesterol (LDL-C), and weight loss, whereas *Butyricimonas* correlates negatively with HDL-C (Shen et al., 2019; Steinert et al., 2020). Postoperative decrease in BMI is significantly and inversely associated with *Faecalibacterium, Lachnospira*, and *Acidaminococcus*. The reduction in body fat mass is closely associated with *Bilophila, Enterococcus*, and *Anaerostipes*, which is directly correlated with hunger levels. A reduced desire to consume sweet foods after surgery inversely correlates with changes in the abundance of *Bulleidia*. Thus, circulating biomarkers and weight loss after BS in patients with NAFLD might be predicted by postsurgical gut microbial changes. In contrast, several bacterial functional metabolic pathways significantly differ after BS. The gut microbiome has a decreased capacity for bacterial toxin production as well as amino acid and carbohydrate metabolism, whereas aminoacyl-transfer-RNA biosynthesis, degradation of aromatic compounds, tyrosine metabolism, and glutathione metabolism pathways are upregulated after RYGB (Murphy et al., 2017; Sánchez-Alcoholado et al., 2019; Dang et al., 2022). Thus, combined proteomic and metabolomic findings have indicated that the postsurgical gut environment generates less energy and better redox and redox counterbalance systems, which might contribute to NAFLD remission (Sanchez-Carrillo et al., 2021).

#### Gut microbial metabolites after bariatric surgery

Gut microbial metabolites are also involved in NAFLD pathogenesis. With changes in the gut microbiome after BS, altered metabolite production contributes to host metabolic improvement in patients with NAFLD after BS. An increased abundance of phylotypes produces SCFAs and branched-chain fatty acids (BCFAs) that are involved in the production of vitamins and fermentation of carbohydrates and proteins in patients after RYGB. Microbial phylotypes enriched after RYGB, such as *Veillonella, Prevotella, Escherichia*, and *Streptococcus*, convert amino acids and carbohydrates into SCFAs and BCFAs, which explains the increased fecal concentrations of butyrate, propionate, and BCFAs (Ilhan et al., 2017). SCFAs can protect the gut barrier and improve stability by reducing LPS translocation into the blood circulation and alleviating liver injury. Furthermore, higher ratios of butyrate and propionate to acetate among the SCFAs in feces after RYGB compared with the baseline value indicate a shift in microbial metabolism from acetate to butyrate and propionate production. As signal-free fatty acid receptors, butyrate with propionate induces a satiety response in the mouse brain, which contributes to weight loss and NAFLD alleviation after RYGB (Lin et al., 2012).

Serum levels of branched-chain amino acids (BCAAs) and the aromatic amino acids (AAAs) isoleucine, leucine, and tyrosine are greatly decreased after BS. Concentrations of metabolites associated with energy metabolism, such as alanine, glucose, and mannose, are also decreased (Han et al., 2022). Reduced levels of BCAAs and glucose are related to a decreased abundance of *Roseburia, R. faecis*, and *D. longicatena*, which are all positively associated with obesity-related microbial dysbiosis (Duncan et al., 2007). In addition, increased serum glycine concentrations with anti-obesity effects are remarkable after BS. Glycine metabolites are positively associated with the gut microbiota, such as *Actinobacteria* and *Bifidobacteria*, which contribute to the amelioration of obesity-related metabolic disorders. Changes in the intestinal environment are associated with altered circulating glycine concentrations in obese Korean patients (Shin et al., 2019). Plasma levels of the bacterial indole derivative indole-3-propionic acid (IPA) and tryptamine also increase after RYGB, and this improves intestinal permeability in human intestinal epithelial cell monolayers (Jennis et al., 2018).

Primary and secondary BAs in feces are both diminished after RYGB, and their abundance are similar to those in individuals of normal weight. A co-occurrence network analysis of fecal microbial phylotypes at the genus level and BAs has shown that enriched microbial phylotypes, *Fusobacterium, Veillonella, Enterococcus, and Akkermansia* after RYGB and *Streptococcus* negatively correlate with various BAs in feces, such as tauroursodeoxycholic acid, LCA, and taurochenodeoxycholic acid (Ilhan et al., 2020). Plasma levels of total BAs are increased and PBA/SBA ratios are decreased at 1 and 5 years after RYGB and SG (Dutia et al., 2015; Risstad et al., 2017; Sung et al., 2018; Ben Izhak et al., 2021). Both GCA and Taurocholate acid (TCA) are decreased, whereas glycoursodeoxycholic acid (GUDCA) is increased in patients after LSG, and these are related to inflammatory cytokines and markers of liver injury (Belgaumkar et al., 2016). GUDCA is a UDCA metabolite that reduces liver steatosis in rat models of NASH and might have the potential to treat NAFLD (Buko et al., 2011). The hepatic BA synthesis inhibitor fibroblast growth factor (FGF)-19 is notably elevated after BS, indicating increased activity of ileal FXR (Jansen et al., 2011). Increased FGF-19 levels are associated with improved hepatic steatosis and lipid profiles (Potthoff et al., 2011). Given the connection between BAs and gut microbiota, postoperative microbial species better suited to the conversion of PBA into SBA might lead to the passive absorption of SBA, changes in the activation of FXR and TGR5, and metabolic improvement (Park et al., 2016; Talavera-Urquijo et al., 2020).

Levels of metabolites are associated with clinical parameters after BS. Reduced circulating glutamate concentrations after SG are associated with improved hyperglycemia, insulin resistance, serum leptin concentrations, and inflammation markers (Liu et al., 2017). Total basal plasma BA concentrations, BMI, and total cholesterol correlate in patients after RYGB and SG (Steinert et al., 2013; Risstad et al., 2017). In addition, glucose and HDL-C are closely associated with secondary bile acids (Ocaña-Wilhelmi et al., 2021). Branched-chain amino acids, glucose, and mannose positively correlate with total cholesterol, LDL-C, and triglycerides, which are related to lipid metabolism (Han et al., 2022).

However, levels of some metabolites associated with inflammation also increase after BS. Increased urinary and serum concentrations of TMAO positively correlate with *Enterobacter, Escherichia, Shigella*, and *Klebsiella* (Li et al., 2021). The abundance of pro-inflammatory fecal LPS and flagellin is increased after RYGB owing to an increase in *Proteobacteria*. Nevertheless, increased levels of antibodies against LPS and flagellin and systemic IgA against LPS might play roles in maintaining the intestinal barrier function after BS. Serum concentrations of LPS decrease after BS, possibly due to decreased gut permeability (Monte et al., 2012).

### Causality studies

Gut microbiota dysbiosis is an important mechanism underlying obesity and NAFLD. Although gut microorganisms associated with the resolution of NAFLD after BS have been identified, confirming a causal link between the gut microbiota and diseases remains challenging. A proposed general strategy to investigate evidence connecting the microbiome to human diseases has been applied in microbiota-related studies of patients with NAFLD undergoing BS (Chaudhari et al., 2021b).

The phenotypes of humans, mice, and GF mice treated with antibiotics are altered during the first stage of the strategy. Mice undergoing vertical sleeve gastrectomy treated with antibiotics develop increased subcutaneous adiposity and reduced alpha diversity of the microbiota, proving that the gut microbiome plays an important role in the effects of BS (Jahansouz et al., 2019). Also, microbial depletion by antibiotics attenuated weight loss and metabolic improvement following RYGB in obese mice (Münzker et al., 2022). During the second stage, phenotypes are transferred using FMTs. Applying FMTs from donor rats that were fed with an HFD and underwent DJB attenuated hepatic steatosis in HFD-fed rats without DJB (Fouladi et al., 2019). GF mice colonized with stools from patients after RYGB and VBG gained less fat mass. A lower respiratory quotient, indicating decreased utilization of carbohydrates as fuel, is also been found in these mice (Liou et al., 2013; Tremaroli et al., 2015). In addition, mice gained more weight when colonized with gut microbiota from patients who did not lose weight after BS compared with those who did (Fouladi et al., 2019). The altered gut microbiota was sufficient to induce decreased host weight and adiposity, which indicated a causal link between the gut microbiota and the effects of BS. During the third and fourth stages, the microbial strains and molecules produce a phenotype. The amount of LCA increases in murine portal veins after SG, vitamin D receptors are activated, and the production of gut-restricted TGR5 agonist cholic acid-7-sulfate (CA7S) is induced (Chaudhari et al., 2021a). Higher levels of CA7S increase GLP-1 secretion in human enteroendocrine cells, which provides a mechanistic link between BA alterations and the metabolic improvement of SG (Chaudhari et al., 2021a). Moreover, weight loss and metabolic improvement benefiting from RYGB microbiota transfer were compromised in intestine-specific FXR inhibitor-treated and Tgr5^–/–^ mice, indicating that intestinal FXR and systemic TGR5 are critical molecular targets for RYGB microbiota transfer in protecting against adiposity and metabolic (Münzker et al., 2022).

In conclusion, increasing evidence indicates that NAFLD improvement after BS correlates and is causally linked to the gut microbiome. Further investigation is needed to elucidate the causal relationship between NAFLD resolution and gut microbiome after BS.

## Future perspectives and conclusion

Changes in the gut microbiome and related metabolites in patients with NAFLD before and after BS have been investigated (Table 3). However, contradictions have been inevitable due to interventions, samples, study population, time points after surgery, and other factors that influence research results (Hoozemans et al., 2021). Accurate experimental designs should include specific sample sizes and validated results. In addition, metabolic diseases and lifestyle factors, such as smoking, that can lead to gut microbiome dysbiosis, underlying diseases, and personal history, should all be considered in NAFLD- and BS-related gut microbial investigations (Aron-Wisnewsky et al., 2020; Lee G. et al., 2020). More objective and rigorous studies are required to confirm the microbiome signatures in NAFLD before and after BS.

**TABLE 3:** Alteration in human microbiome and related metabolites in NAFLD and after bariatric surgery.

	Gut microbiome		Gut microbial metabolites	
	Increased abundance	Decreased abundance	Increased abundance	Decreased abundance
NAFLD	*Bacteroidetes, Proteobacteria, Dorea, Lactobacillus, Clostridium, Ruminococcus, Prevotella*	*Firmicutes, Lactobacillales, Lachnospiraceae, Ruminococcaceae, Lactobacillaceae, Veillonellaceae*	SCFA, Total fecal BAs, PBA/SBA ratio, DCA, TMAO, endogenous ethanol, LPS	Choline, indole
BS	*Bacteroidetes, Fusobacteria, Verrucomicrobia, Proteobacteria, Lactobacillales, Enterococcus, Gammaproteobacteria, Akkermansia, Enterobacteriales;* Anaerobes, such as *Clostridium* become more abundant in SG whereas aero-tolerant bacteria such as *Streptococcus* and *Veillonella*, become more abundant after RYGB	*Firmicutes, Clostridiales, Clostridiaceae, Blautia*, and *Dorea*	BCFAs, butyrate, propionate, total plasma BAs, GUDCA, indole-3-propionic acid (IPA)	BCAAs, AAAs, PBA/SBA ratios, GCA, TCA, acetate, plasma LPS

NAFLD, non-alcoholic fatty liver disease; BS, bariatric surgery; RYGB, Roux-en-Y gastric bypass; SG, sleeve gastrectomy; AAA, aromatic amino acid; BCAA, branched-chain amino acid; TCA, taurocholate acid; GUDCA, glycoursodeoxycholic acid; DCA, deoxycholic; SCFAs, short-chain fatty acids; PBA, primary bile acid; SBAs, secondary bile acid; GCA, glycocholic acids; TMAO, trimethylamine-N-oxide; BCFAs, branched-chain fatty acids; LPS, lipopolysaccharides.

Although the gut microbiota is a promising research topic, some major concerns require emphasis. The results of most studies on the gut microbiomes of patients with NAFLD correlate, and the involvement of gut microbiome-related mechanisms in other metabolic diseases, such as type 2 diabetes, has recently been revealed (Ridlon et al., 2006; Ridaura et al., 2013; Müller et al., 2019). However, the specific functions and mechanisms of action of bacterial strains and molecules in NAFLD and BS have not been explored in detail. Further investigation into the molecular mechanisms linking functional microbiomes and microbial metabolites to NAFLD and BS is needed to define the roles of the gut microbiome (Cerreto et al., 2021). Given the functions of the gut microbiome in NAFLD and BS, achieving clinical translation is important. Manipulation of the microbiome to treat diseases and improve the effects of BS is a concern (Rossell et al., 2020). The value of FMT has been clinically validated, and it can improve the metabolism of recipients after BS (Fouladi et al., 2019). Microbiome-directed therapies beyond FMT include commensal bacteria, microbial consortia, food, prebiotics, engineered symbiotic bacteria, and microbiota-derived proteins and metabolites aimed at reconstituting or altering the intestinal microbiome with specific bacterial species (Sorbara and Pamer, 2022). A controlled diet after BS has proven beneficial to the recovery of a healthy body weight after BS (Rossell et al., 2020). Probiotic *Lactobacillus bulgaricus*, *Lactobacillus helveticus*, and *Pediococcus pentosaceus KID7* alleviate steatohepatitis in mice fed with an HFD by modulating the gut microbiota composition and inflammatory pathways involved in the gut-liver axis of NAFLD (Lee N. Y. et al., 2020). However, all therapies face considerable challenges and follow a long and clinical treacherous path. Preclinical and clinical studies are required before microbiome-related therapy can become optimized.

In conclusion, changes in the gut microbiome are related to NAFLD pathogenesis and development. The most effective treatment is BS, resulting in significant weight loss in patients with obesity and related diseases, as it alleviates hepatic steatosis and improves host metabolism in patients with NAFLD. This surgery also leads to modifications of the gut microbiota. Changes in the gut microbiome and related metabolites are associated with NAFLD resolution after BS. However, the mechanisms underlying how the gut microbiome improves NAFLD after BS remain unclear. Achieving clinical translation based on microbiota-related mechanisms remains challenging. Thus, future investigations should focus on these targets to learn more about the composition and function of the microbiota to benefit patients.

## Author contributions

YX conceptualized the review and wrote the manuscript. MR, JY, CC, WC, XZ, and DL provided significant suggestions in the text. FJ provided the financial support. All authors have approved of the publication of the manuscript.

## References

[B1] Adams L. A. Wang Z. Liddle C. Melton P. E. Ariff A. Chandraratna H. (2020). Bile acids associate with specific gut microbiota, low-level alcohol consumption and liver fibrosis in patients with non-alcoholic fatty liver disease. *Liver Int.* 40 1356–1365. 10.1111/liv.14453 32243703

[B2] Albillos A. de Gottardi A. Rescigno M. (2020). The gut-liver axis in liver disease: Pathophysiological basis for therapy. *J. Hepatol.* 72 558–577. 10.1016/j.jhep.2019.10.003 31622696

[B3] Angelini G. Castagneto G. L. Del Corpo G. Giordano C. Cerbelli B. Severino A. (2019). New insight into the mechanisms of ectopic fat deposition improvement after bariatric surgery. *Sci. Rep.* 9:17315. 10.1038/s41598-019-53702-4 31754142 PMC6872729

[B4] Angelini G. Castagneto-Gissey L. Casella-Mariolo J. Caristo M. E. Russo M. F. Lembo E. (2020). Duodenal-jejunal bypass improves nonalcoholic fatty liver disease independently of weight loss in rodents with diet-induced obesity. *Am. J. Physiol. Gastrointest. Liver Physiol.* 319 G502–G511. 10.1152/ajpgi.00357.2019 32812775

[B5] Aragonès G. Colom-Pellicer M. Aguilar C. Guiu-Jurado E. Martínez S. Sabench F. (2020). Circulating microbiota-derived metabolites: A liquid biopsy? *Int. J. Obes.* 44 875–885. 10.1038/s41366-019-0430-0 31388096 PMC7101279

[B6] Aron-Wisnewsky J. Vigliotti C. Witjes J. Le P. Holleboom A. G. Verheij J. (2020). Gut microbiota and human NAFLD: Disentangling microbial signatures from metabolic disorders. *Nat. Rev. Gastroenterol. Hepatol.* 17 279–297. 10.1038/s41575-020-0269-9 32152478

[B7] Baker S. S. Baker R. D. Liu W. Nowak N. J. Zhu L. (2010). Role of alcohol metabolism in non-alcoholic steatohepatitis. *PLoS One* 5:e9570. 10.1371/journal.pone.0009570 20221393 PMC2833196

[B8] Barrea L. Annunziata G. Muscogiuri G. Di Somma C. Laudisio D. Maisto M. (2018). Trimethylamine-N-oxide (TMAO) as novel potential biomarker of early predictors of metabolic syndrome. *Nutrients* 10:1971. 10.3390/nu10121971 30551613 PMC6316855

[B9] Behary J. Amorim N. Jiang X. Raposo A. Gong L. McGovern E. (2021). Gut microbiota impact on the peripheral immune response in non-alcoholic fatty liver disease related hepatocellular carcinoma. *Nat. Commun.* 12:187. 10.1038/s41467-020-20422-7 33420074 PMC7794332

[B10] Belgaumkar A. P. Vincent R. P. Carswell K. A. Hughes R. D. Alaghband-Zadeh J. Mitry R. R. (2016). Changes in bile acid profile after laparoscopic sleeve gastrectomy are associated with improvements in metabolic profile and fatty liver disease. *Obes. Surg.* 26 1195–1202. 10.1007/s11695-015-1878-1 26337697

[B11] Bellahcene M. O’Dowd J. F. Wargent E. T. Zaibi M. S. Hislop D. C. Ngala R. A. (2013). Male mice that lack the G-protein-coupled receptor GPR41 have low energy expenditure and increased body fat content. *Br. J. Nutr.* 109 1755–1764. 10.1017/S0007114512003923 23110765

[B12] Ben Izhak M. Eshel A. Cohen R. Madar-Shapiro L. Meiri H. Wachtel C. (2021). Projection of gut microbiome pre- and post-bariatric surgery to predict surgery outcome. *mSystems* 6:e0136720. 10.1128/mSystems.01367-20 34100636 PMC8269264

[B13] Boursier J. Mueller O. Barret M. Machado M. Fizanne L. Araujo-Perez F. (2016). The severity of nonalcoholic fatty liver disease is associated with gut dysbiosis and shift in the metabolic function of the gut microbiota. *Hepatology* 63 764–775. 10.1002/hep.28356 26600078 PMC4975935

[B14] Brun P. Castagliuolo I. Leo V. D. Buda A. Pinzani M. Palù G. (2007). Increased intestinal permeability in obese mice: New evidence in the pathogenesis of nonalcoholic steatohepatitis. *Am. J. Physiol. Gastrointest. Liver Physiol.* 292 G518–G525. 10.1152/ajpgi.00024.2006 17023554

[B15] Buko V. U. Kuzmitskaya-Nikolaeva I. A. Naruta E. E. Lukivskaya O. Y. Kirko S. N. Tauschel H. (2011). Ursodeoxycholic acid dose-dependently improves liver injury in rats fed a methionine- and choline-deficient diet. *Hepatol. Res.* 41 647–659. 10.1111/j.1872-034X.2011.00820.x 21711424

[B16] Bult M. J. F. van Dalen T. Muller A. F. (2008). Surgical treatment of obesity. *Eur. J. Endocrinol.* 158 135–145. 18230819 10.1530/EJE-07-0145

[B17] Byrne C. D. Targher G. (2016). EASL–EASD–EASO clinical practice guidelines for the management of non-alcoholic fatty liver disease: Is universal screening appropriate? *Diabetologia* 59 1141–1144. 10.1007/s00125-016-3910-y 27053232

[B18] Carpino G. Del Ben M. Pastori D. Carnevale R. Baratta F. Overi D. (2020). Increased liver localization of lipopolysaccharides in human and experimental NAFLD. *Hepatology* 72 470–485. 10.1002/hep.31056 31808577

[B19] Carr R. M. Reid A. E. (2015). FXR agonists as therapeutic agents for non-alcoholic fatty liver disease. *Curr. Atheroscler. Rep.* 17:500. 10.1007/s11883-015-0500-2 25690590

[B20] Caussy C. Tripathi A. Humphrey G. Bassirian S. Singh S. Faulkner C. (2019). A gut microbiome signature for cirrhosis due to nonalcoholic fatty liver disease. *Nat. Commun.* 10:1406. 30926798 10.1038/s41467-019-09455-9 PMC6440960

[B21] Cerreto M. Santopaolo F. Gasbarrini A. Pompili M. Ponziani F. (2021). Bariatric surgery and liver disease: General considerations and role of the gut–liver axis. *Nutrients* 13:2649. 10.3390/nu13082649 34444807 PMC8399840

[B22] Chalasani N. Younossi Z. Lavine J. E. Charlton M. Cusi K. Rinella M. (2018). The diagnosis and management of nonalcoholic fatty liver disease: Practice guidance from the American Association for the study of liver diseases. *Hepatology* 67 328–357. 10.1002/hep.29367 28714183

[B23] Chaudhari S. N. Luo J. N. Harris D. A. Aliakbarian H. Yao L. Paik D. (2021a). A microbial metabolite remodels the gut-liver axis following bariatric surgery. *Cell Host Microbe* 29 408–424.e7. 10.1016/j.chom.2020.12.004 33434516 PMC7954942

[B24] Chaudhari S. N. McCurry M. D. Devlin A. S. (2021b). Chains of evidence from correlations to causal molecules in microbiome-linked diseases. *Nat. Chem. Biol.* 17 1046–1056. 10.1038/s41589-021-00861-z 34552222 PMC8480537

[B25] Chen F. Esmaili S. Rogers G. B. Bugianesi E. Petta S. Marchesini G. (2020). Lean NAFLD: A distinct entity shaped by differential metabolic adaptation. *Hepatology* 71 1213–1227. 10.1002/hep.30908 31442319

[B26] Chen X. Zhang Z. Li H. Zhao J. Wei X. Lin W. (2020). Endogenous ethanol produced by intestinal bacteria induces mitochondrial dysfunction in non-alcoholic fatty liver disease. *J. Gastroenterol. Hepatol.* 35 2009–2019. 10.1111/jgh.15027 32150306

[B27] Chen Y. Liu Y. Zhou R. Chen X. Wang C. Tan X. (2016). Associations of gut-flora-dependent metabolite trimethylamine-N-oxide, betaine and choline with non-alcoholic fatty liver disease in adults. *Sci. Rep.* 6:19076. 10.1038/srep19076 26743949 PMC4705470

[B28] Cho C. E. Taesuwan S. Malysheva O. V. Bender E. Tulchinsky N. F. Yan J. (2017). Trimethylamine-N-oxide (TMAO) response to animal source foods varies among healthy young men and is influenced by their gut microbiota composition: A randomized controlled trial. *Mol. Nutr. Food Res.* 61:1600324. 10.1002/mnfr.201600324 27377678

[B29] Compare D. Rocco A. Sanduzzi Z. M. Nardone G. (2016). The gut bacteria-driven obesity development. *Dig. Dis.* 34 221–229. 10.1159/000443356 27028448

[B30] Corbin K. D. Zeisel S. H. (2012). Choline metabolism provides novel insights into nonalcoholic fatty liver disease and its progression. *Curr. Opin. Gastroenterol.* 28 159–165. 10.1097/MOG.0b013e32834e7b4b 22134222 PMC3601486

[B31] Cornejo-Pareja I. Molina-Vega M. Gómez-Pérez A. M. Damas-Fuentes M. Tinahones F. J. (2021). Factors related to weight loss maintenance in the medium-long term after bariatric surgery: A review. *J. Clin. Med.* 10:1739. 10.3390/jcm10081739 33923789 PMC8073104

[B32] Cotter T. G. Rinella M. (2020). Nonalcoholic fatty liver disease 2020: The state of the disease. *Gastroenterology* 158 1851–1864. 10.1053/j.gastro.2020.01.052 32061595

[B33] Craven L. Rahman A. Nair Parvathy S. Beaton M. Silverman J. Qumosani K. (2020). Allogenic fecal microbiota transplantation in patients with nonalcoholic fatty liver disease improves abnormal small intestinal permeability: A randomized control trial. *Am. J. Gastroenterol.* 115 1055–1065. 10.14309/ajg.0000000000000661 32618656

[B34] Cummings D. E. Rubino F. (2018). Metabolic surgery for the treatment of type 2 diabetes in obese individuals. *Diabetologia* 61 257–264. 29224190 10.1007/s00125-017-4513-y PMC6448954

[B35] Dang J. T. Mocanu V. Park H. Laffin M. Hotte N. Karmali S. (2022). Roux-en-Y gastric bypass and sleeve gastrectomy induce substantial and persistent changes in microbial communities and metabolic pathways. *Gut Microbes* 14:2050636. 10.1080/19490976.2022.2050636 35316158 PMC8942407

[B36] Dar M. S. Chapman W. H. Pender J. R. Drake A. J. O’Brien K. Tanenberg R. J. (2012). GLP-1 response to a mixed meal: What happens 10 years after Roux-en-Y gastric bypass (RYGB)? *Obes. Surg.* 22 1077–1083. 10.1007/s11695-012-0624-1 22419108

[B37] Davies N. K. O’Sullivan J. M. Plank L. D. Murphy R. (2019). Altered gut microbiome after bariatric surgery and its association with metabolic benefits: A systematic review. *Surg. Obes. Relat. Dis.* 15 656–665. 30824335 10.1016/j.soard.2019.01.033

[B38] de Brito E. S. M. Tustumi F. de Miranda N. A. Dantas A. Santo M. A. Cecconello I. (2021). Gastric bypass compared with sleeve gastrectomy for nonalcoholic fatty liver disease: A systematic review and meta-analysis. *Obes. Surg.* 31 2762–2772. 10.1007/s11695-021-05412-y 33846949

[B39] de Jonge C. Fuentes S. Zoetendal E. G. Bouvy N. D. Nelissen R. Buurman W. A. (2019). Metabolic improvement in obese patients after duodenal–jejunal exclusion is associated with intestinal microbiota composition changes. *Int. J. Obes.* 43 2509–2517. 10.1038/s41366-019-0336-x 30765893

[B40] De Munck T. Xu P. Verwijs H. Masclee A. Jonkers D. Verbeek J. (2020). Intestinal permeability in human nonalcoholic fatty liver disease: A systematic review and meta-analysis. *Liver Int.* 40 2906–2916. 10.1111/liv.14696 33037768 PMC7756870

[B41] Del Chierico F. Nobili V. Vernocchi P. Russo A. De Stefanis C. Gnani D. (2016). Gut microbiota profiling of pediatric nonalcoholic fatty liver disease and obese patients unveiled by an integrated meta-omics-based approach. *Hepatology* 65 451–464. 10.1002/hep.28572 27028797

[B42] Demir M. Lang S. Hartmann P. Duan Y. Martin A. Miyamoto Y. (2022). The fecal mycobiome in non-alcoholic fatty liver disease. *J. Hepatol.* 76 788–799. 10.1016/j.jhep.2021.11.029 34896404 PMC8981795

[B43] Duarte S. Stefano J. T. Miele L. Ponziani F. R. Souza-Basqueira M. Okada L. (2018). Gut microbiome composition in lean patients with NASH is associated with liver damage independent of caloric intake: A prospective pilot study. *Nutr. Metab. Cardiovasc. Dis.* 28 369–384. 10.1016/j.numecd.2017.10.014 29482963

[B44] Dumas M. Barton R. H. Toye A. Cloarec O. Blancher C. Rothwell A. (2006). Metabolic profiling reveals a contribution of gut microbiota to fatty liver phenotype in insulin-resistant mice. *Proc. Natl. Acad. Sci. U.S.A.* 103 12511–12516. 10.1073/pnas.0601056103 16895997 PMC1567909

[B45] Duncan S. H. Belenguer A. Holtrop G. Johnstone A. M. Flint H. J. Lobley G. E. (2007). Reduced dietary intake of carbohydrates by obese subjects results in decreased concentrations of butyrate and butyrate-producing bacteria in feces. *Appl. Environ. Microbiol.* 73 1073–1078. 10.1128/AEM.02340-06 17189447 PMC1828662

[B46] Dutia R. Embrey M. O’Brien S. Haeusler R. A. Agénor K. K. Homel P. (2015). Temporal changes in bile acid levels and 12α-hydroxylation after Roux-en-Y gastric bypass surgery in type 2 diabetes. *Int. J. Obes.* 39 806–813. 10.1038/ijo.2015.1 PMC4422767 25599611

[B47] Eckburg P. B. Bik E. M. Bernstein C. N. Purdom E. Dethlefsen L. Sargent M. (2005). Diversity of the human intestinal microbial flora. *Science* 308 1635–1638. 10.1126/science.1110591 15831718 PMC1395357

[B48] Engstler A. J. Aumiller T. Degen C. Dürr M. Weiss E. Maier I. B. (2016). Insulin resistance alters hepatic ethanol metabolism: Studies in mice and children with non-alcoholic fatty liver disease. *Gut* 65 1564–1571. 10.1136/gutjnl-2014-308379 26006114

[B49] Estes C. Anstee Q. M. Arias-Loste M. T. Bantel H. Bellentani S. Caballeria J. (2018). Modeling NAFLD disease burden in China, France, Germany, Italy, Japan, Spain, United Kingdom, and United States for the period 2016–2030. *J. Hepatol.* 69 896–904. 10.1016/j.jhep.2018.05.036 29886156

[B50] Falony G. Vieira-Silva S. Raes J. (2015). Microbiology meets big data: The case of gut microbiota–derived trimethylamine. *Annu. Rev. Microbiol.* 69 305–321. 10.1146/annurev-micro-091014-104422 26274026

[B51] Farin W. Oñate F. P. Plassais J. Bonny C. Beglinger C. Woelnerhanssen B. (2020). Impact of laparoscopic Roux-en-Y gastric bypass and sleeve gastrectomy on gut microbiota: A metagenomic comparative analysis. *Surg. Obes. Relat. Dis.* 16 852–862. 10.1016/j.soard.2020.03.014 32360114

[B52] Fei N. Bruneau A. Zhang X. Wang R. Wang J. Rabot S. (2020). Endotoxin producers overgrowing in human gut microbiota as the causative agents for nonalcoholic fatty liver disease. *mBio* 11:e03263-19. 10.1128/mBio.03263-19 32019793 PMC7002352

[B53] Festi D. Schiumerini R. Eusebi L. H. Marasco G. Taddia M. Colecchia A. (2014). Gut microbiota and metabolic syndrome. *World J. Gastroenterol.* 20 16079–16094. 25473159 10.3748/wjg.v20.i43.16079 PMC4239493

[B54] Fouladi F. Brooks A. E. Fodor A. A. Carroll I. M. Bulik-Sullivan E. C. Tsilimigras M. C. B. (2019). The role of the gut microbiota in sustained weight loss following Roux-en-Y gastric bypass surgery. *Obes. Surg.* 29 1259–1267. 10.1007/s11695-018-03653-y 30604078

[B55] Fuchs C. Traussnigg S. Trauner M. (2016). Nuclear receptor modulation for the treatment of nonalcoholic fatty liver disease. *Semin. Liver Dis.* 36 069–086. 10.1055/s-0036-1571296 26870934

[B56] Fukuda N. Ojima T. Hayata K. Katsuda M. Kitadani J. Takeuchi A. (2022). Laparoscopic sleeve gastrectomy for morbid obesity improves gut microbiota balance, increases colonic mucosal-associated invariant T cells and decreases circulating regulatory T cells. *Surg. Endosc.* 10.1007/s00464-022-09122-z [Epub ahead of print]. 35182212

[B57] Gao X. Liu X. Xu J. Xue C. Xue Y. Wang Y. (2014). Dietary trimethylamine N-oxide exacerbates impaired glucose tolerance in mice fed a high fat diet. *J. Biosci. Bioeng.* 118 476–481. 10.1016/j.jbiosc.2014.03.001 24721123

[B58] Groschwitz K. R. Hogan S. P. (2009). Intestinal barrier function: Molecular regulation and disease pathogenesis. *J. Allergy Clin. Immunol.* 124 3–20; quiz 21–22. 19560575 10.1016/j.jaci.2009.05.038 PMC4266989

[B59] Guarner F. Malagelada J. (2003). Gut flora in health and disease. *Lancet* 361 512–519. 12583961 10.1016/S0140-6736(03)12489-0

[B60] Guo Y. Huang Z. P. Liu C. Q. Qi L. Sheng Y. Zou D. J. (2018). Modulation of the gut microbiome: A systematic review of the effect of bariatric surgery. *Eur. J. Endocrinol.* 178 43–56. 10.1530/EJE-17-0403 28916564

[B61] Han Y. Kim G. Ahn E. Jung S. Jung Y. Kim Y. (2022). Integrated metagenomics and metabolomics analysis illustrates the systemic impact of the gut microbiota on host metabolism after bariatric surgery. *Diabetes Obes. Metab.* 24 1224–1234. 10.1111/dom.14689 35257467 PMC9313881

[B62] Hoozemans J. de Brauw M. Nieuwdorp M. Gerdes V. (2021). Gut microbiome and metabolites in patients with NAFLD and after bariatric surgery: A comprehensive review. *Metabolites* 11:353. 10.3390/metabo11060353 34072995 PMC8227414

[B63] Hotamisligil G. S. Murray D. L. Choy L. N. Spiegelman B. M. (1994). Tumor necrosis factor alpha inhibits signaling from the insulin receptor. *Proc. Natl. Acad. Sci. U.S.A.* 91 4854–4858. 8197147 10.1073/pnas.91.11.4854 PMC43887

[B64] Huang D. Q. El-Serag H. B. Loomba R. (2021). Global epidemiology of NAFLD-related HCC: Trends, predictions, risk factors and prevention. *Nat. Rev. Gastroenterol. Hepatol.* 18 223–238. 10.1038/s41575-020-00381-6 33349658 PMC8016738

[B65] Hutch C. R. Sandoval D. (2017). The role of GLP-1 in the metabolic success of bariatric surgery. *Endocrinology* 158 4139–4151. 29040429 10.1210/en.2017-00564 PMC5711387

[B66] Ilhan Z. E. DiBaise J. K. Dautel S. E. Isern N. G. Kim Y. Hoyt D. W. (2020). Temporospatial shifts in the human gut microbiome and metabolome after gastric bypass surgery. *NPJ Biofilms Microbiomes* 6:12. 10.1038/s41522-020-0122-5 32170068 PMC7070067

[B67] Ilhan Z. E. DiBaise J. K. Isern N. G. Hoyt D. W. Marcus A. K. Kang D. (2017). Distinctive microbiomes and metabolites linked with weight loss after gastric bypass, but not gastric banding. *ISME J.* 11 2047–2058. 10.1038/ismej.2017.71 28548658 PMC5563958

[B68] Jahansouz C. Staley C. Kizy S. Xu H. Hertzel A. V. Coryell J. (2019). Antibiotic-induced disruption of intestinal microbiota contributes to failure of vertical sleeve gastrectomy. *Ann. Surg.* 269 1092–1100. 10.1097/SLA.0000000000002729 31082907

[B69] Jansen P. L. M. van Werven J. Aarts E. Berends F. Janssen I. Stoker J. (2011). Alterations of hormonally active fibroblast growth factors after Roux-en-Y gastric bypass surgery. *Dig. Dis.* 29 48–51. 21691104 10.1159/000324128

[B70] Jennis M. Cavanaugh C. R. Leo G. C. Mabus J. R. Lenhard J. Hornby P. J. (2018). Microbiota-derived tryptophan indoles increase after gastric bypass surgery and reduce intestinal permeability in vitro and in vivo. *Neurogastroenterol. Motil.* 30:e13178. 10.1111/nmo.13178 28782205

[B71] Jiang W. Wu N. Wang X. Chi Y. Zhang Y. Qiu X. (2015). Dysbiosis gut microbiota associated with inflammation and impaired mucosal immune function in intestine of humans with non-alcoholic fatty liver disease. *Sci. Rep.* 5:8096. 25644696 10.1038/srep08096 PMC4314632

[B72] Jiao N. Baker S. S. Chapa-Rodriguez A. Liu W. Nugent C. A. Tsompana M. (2018). Suppressed hepatic bile acid signalling despite elevated production of primary and secondary bile acids in NAFLD. *Gut* 67 1881–1891. 10.1136/gutjnl-2017-314307 28774887

[B73] Kawai T. Akira S. (2009). The roles of TLRs, RLRs and NLRs in pathogen recognition. *Int. Immunol.* 21 317–337. 10.1093/intimm/dxp017 19246554 PMC2721684

[B74] Kawai T. Akira S. (2010). The role of pattern-recognition receptors in innate immunity: Update on Toll-like receptors. *Nat. Immunol.* 11 373–384. 10.1038/ni.1863 20404851

[B75] Kim H. Joo E. Cheong H. S. Kim Y. Kim H. Shin H. (2019). Gut microbiota and risk of persistent nonalcoholic fatty liver diseases. *J. Clin. Med.* 8:1089. 10.3390/jcm8081089 PMC6722749 31344854

[B76] Kolodziejczyk A. A. Zheng D. Shibolet O. Elinav E. (2019). The role of the microbiome in NAFLD and NASH. *EMBO Mol. Med.* 11:e9302. 30591521 10.15252/emmm.201809302 PMC6365925

[B77] Krawczyk M. Bonfrate L. Portincasa P. (2010). Nonalcoholic fatty liver disease. *Best Pract. Res. Clin. Gastroenterol.* 24 695–708. 10.1016/j.bpg.2010.08.005 20955971

[B78] Lassailly G. Caiazzo R. Buob D. Pigeyre M. Verkindt H. Labreuche J. (2015). Bariatric surgery reduces features of nonalcoholic steatohepatitis in morbidly obese patients. *Gastroenterology* 149 379–388; quiz e15–e16. 25917783 10.1053/j.gastro.2015.04.014

[B79] Lassailly G. Caiazzo R. Ntandja-Wandji L. C. Gnemmi V. Baud G. Verkindt H. (2020). Bariatric surgery provides long-term resolution of nonalcoholic steatohepatitis and regression of fibrosis. *Gastroenterology* 159 1290–1301.e5. 10.1053/j.gastro.2020.06.006 32553765

[B80] Lee C. J. Florea L. Sears C. L. Maruthur N. Potter J. J. Schweitzer M. (2019a). Changes in gut microbiome after bariatric surgery versus medical weight loss in a pilot randomized trial. *Obes. Surg.* 29 3239–3245. 10.1007/s11695-019-03976-4 31256356

[B81] Lee D. H. Park M. H. Hwang C. J. Kim Y. Hwang D. Y. Han S. B. (2019b). Parkin deficiency prevents chronic ethanol-induced hepatic lipid accumulation through β-catenin accumulation. *Cell Commun. Signal.* 17:104. 10.1186/s12964-019-0424-5 31438968 PMC6704582

[B82] Lee Y. Doumouras A. G. Yu J. Brar K. Banfield L. Gmora S. (2019c). Complete resolution of nonalcoholic fatty liver disease after bariatric surgery: A systematic review and meta-analysis. *Clin. Gastroenterol. Hepatol.* 17 1040–1060.e11. 30326299 10.1016/j.cgh.2018.10.017

[B83] Lee G. You H. J. Bajaj J. S. Joo S. K. Yu J. Park S. (2020). Distinct signatures of gut microbiome and metabolites associated with significant fibrosis in non-obese NAFLD. *Nat. Commun.* 11:4982. 10.1038/s41467-020-18754-5 33020474 PMC7536225

[B84] Lee N. Y. Yoon S. J. Han D. H. Gupta H. Youn G. S. Shin M. J. (2020). *Lactobacillus* and *Pediococcus* ameliorate progression of non-alcoholic fatty liver disease through modulation of the gut microbiome. *Gut Microbes* 11 882–899. 10.1080/19490976.2020.1712984 31965894 PMC7524267

[B85] Leung C. Rivera L. Furness J. B. Angus P. W. (2016). The role of the gut microbiota in NAFLD. *Nat. Rev. Gastroenterol. Hepatol.* 13 412–425. 10.1038/nrgastro.2016.85 27273168

[B86] Li J. V. Ashrafian H. Sarafian M. Homola D. Rushton L. Barker G. (2021). Roux-en-Y gastric bypass-induced bacterial perturbation contributes to altered host-bacterial co-metabolic phenotype. *Microbiome* 9:139. 10.1186/s40168-021-01086-x 34127058 PMC8201742

[B87] Li S. Ghoshal S. Sojoodi M. Arora G. Masia R. Erstad D. J. (2019). Pioglitazone reduces hepatocellular carcinoma development in two rodent models of cirrhosis. *J. Gastrointest. Surg.* 23 101–111. 30367397 10.1007/s11605-018-4004-6 PMC6328630

[B88] Lieber C. S. (1999). Microsomal ethanol-oxidizing system (MEOS): The first 30 years (1968-1998)–a review. *Alcohol. Clin. Exp. Res.* 23 991–1007. 10397283

[B89] Lin H. V. Frassetto A. Kowalik E. J. Jr. Nawrocki A. R. Lu M. M. Kosinski J. R. (2012). Butyrate and propionate protect against diet-induced obesity and regulate gut hormones via free fatty acid receptor 3-independent mechanisms. *PLoS One* 7:e35240. 10.1371/journal.pone.0035240 22506074 PMC3323649

[B90] Liou A. P. Paziuk M. Luevano J. Machineni S. Turnbaugh P. J. Kaplan L. M. (2013). Conserved shifts in the gut microbiota due to gastric bypass reduce host weight and adiposity. *Sci. Transl. Med.* 5:178ra141. 10.1126/scitranslmed.3005687 23536013 PMC3652229

[B91] Liu R. Hong J. Xu X. Feng Q. Zhang D. Gu Y. (2017). Gut microbiome and serum metabolome alterations in obesity and after weight-loss intervention. *Nat. Med.* 23 859–868. 28628112 10.1038/nm.4358

[B92] Liu X. Xue R. Ji L. Zhang X. Wu J. Gu J. (2014). Activation of farnesoid X receptor (FXR) protects against fructose-induced liver steatosis via inflammatory inhibition and ADRP reduction. *Biochem. Biophys. Res. Commun.* 450 117–123. 10.1016/j.bbrc.2014.05.072 24875360

[B93] Loomba R. Friedman S. L. Shulman G. I. (2021). Mechanisms and disease consequences of nonalcoholic fatty liver disease. *Cell* 184 2537–2564. 33989548 10.1016/j.cell.2021.04.015 PMC12168897

[B94] Loomba R. Seguritan V. Li W. Long T. Klitgord N. Bhatt A. (2017). Gut microbiome-based metagenomic signature for non-invasive detection of advanced fibrosis in human nonalcoholic fatty liver disease. *Cell Metab.* 25 1054–1062.e5. 28467925 10.1016/j.cmet.2017.04.001 PMC5502730

[B95] Lund M. L. Sorrentino G. Egerod K. L. Kroone C. Mortensen B. Knop F. K. (2020). L-cell differentiation is induced by bile acids through GPBAR1 and paracrine GLP-1 and serotonin signaling. *Diabetes* 69 614–623. 10.2337/db19-0764 32041793 PMC7224989

[B96] Luther J. Garber J. J. Khalili H. Dave M. Bale S. S. Jindal R. (2015). Hepatic injury in nonalcoholic steatohepatitis contributes to altered intestinal permeability. *Cell. Mol. Gastroenterol. Hepatol.* 1 222–232.e2. 26405687 10.1016/j.jcmgh.2015.01.001 PMC4578658

[B97] Mabey J. G. Chaston J. M. Castro D. G. Adams T. D. Hunt S. C. Davidson L. E. (2020). Gut microbiota differs a decade after bariatric surgery relative to a nonsurgical comparison group. *Surg. Obes. Relat. Dis.* 16 1304–1311. 10.1016/j.soard.2020.04.006 32466962 PMC7483956

[B98] Martinez K. B. Pierre J. F. Chang E. B. (2016). The gut microbiota. *Gastroenterol. Clin. N. Am.* 45 601–614. 10.1016/j.gtc.2016.07.001 PMC5127273 27837775

[B99] Martínez-del Campo A. Bodea S. Hamer H. A. Marks J. A. Haiser H. J. Turnbaugh P. J. (2015). Characterization and detection of a widely distributed gene cluster that predicts anaerobic choline utilization by human gut bacteria. *mBio* 6:e00042-15. 10.1128/mBio.00042-15 25873372 PMC4453576

[B100] Mazzini G. S. Khoraki J. Browning M. G. Wu J. Zhou H. Price E. T. (2021). Gastric bypass increases circulating bile acids and activates hepatic farnesoid X receptor (FXR) but requires intact peroxisome proliferator activator receptor alpha (PPARα) signaling to significantly reduce liver fat content. *J. Gastrointest. Surg.* 25 871–879. 10.1007/s11605-021-04908-3 33555523

[B101] Michail S. Lin M. Frey M. R. Fanter R. Paliy O. Hilbush B. (2014). Altered gut microbial energy and metabolism in children with non-alcoholic fatty liver disease. *FEMS Microbiol. Ecol.* 91 1–9. 10.1093/femsec/fiu002 PMC4358749 25764541

[B102] Miele L. Valenza V. La Torre G. Montalto M. Cammarota G. Ricci R. (2009). Increased intestinal permeability and tight junction alterations in nonalcoholic fatty liver disease. *Hepatology* 49 1877–1887. 19291785 10.1002/hep.22848

[B103] Miura K. (2014). Role of gut microbiota and Toll-like receptors in nonalcoholic fatty liver disease. *World J. Gastroenterol.* 20 7381–7391. 24966608 10.3748/wjg.v20.i23.7381 PMC4064083

[B104] Miura K. Kodama Y. Inokuchi S. Schnabl B. Aoyama T. Ohnishi H. (2010). Toll-like receptor 9 promotes steatohepatitis by induction of interleukin-1β in mice. *Gastroenterology* 139 323–334.e7. 10.1053/j.gastro.2010.03.052 20347818 PMC4631262

[B105] Monte S. V. Caruana J. A. Ghanim H. Sia C. L. Korzeniewski K. Schentag J. J. (2012). Reduction in endotoxemia, oxidative and inflammatory stress, and insulin resistance after Roux-en-Y gastric bypass surgery in patients with morbid obesity and type 2 diabetes mellitus. *Surgery* 151 587–593. 10.1016/j.surg.2011.09.038 22088821

[B106] Morrison D. J. Preston T. (2016). Formation of short chain fatty acids by the gut microbiota and their impact on human metabolism. *Gut Microbes* 7 189–200. 26963409 10.1080/19490976.2015.1134082 PMC4939913

[B107] Mridha A. R. Wree A. Robertson A. A. B. Yeh M. M. Johnson C. D. Van Rooyen D. M. (2017). NLRP3 inflammasome blockade reduces liver inflammation and fibrosis in experimental NASH in mice. *J. Hepatol.* 66 1037–1046. 10.1016/j.jhep.2017.01.022 28167322 PMC6536116

[B108] Müller M. Hernández M. Goossens G. H. Reijnders D. Holst J. J. Jocken J. (2019). Circulating but not faecal short-chain fatty acids are related to insulin sensitivity, lipolysis and GLP-1 concentrations in humans. *Sci. Rep.* 9:12515. 31467327 10.1038/s41598-019-48775-0 PMC6715624

[B109] Münzker J. Haase N. Till A. Sucher R. Haange S. B. Nemetschke L. (2022). Functional changes of the gastric bypass microbiota reactivate thermogenic adipose tissue and systemic glucose control via intestinal FXR-TGR5 crosstalk in diet-induced obesity. *Microbiome* 10:96. 10.1186/s40168-022-01264-5 35739571 PMC9229785

[B110] Murphy R. Tsai P. Jüllig M. Liu A. Plank L. Booth M. (2017). Differential changes in gut microbiota after gastric bypass and sleeve gastrectomy bariatric surgery vary according to diabetes remission. *Obes. Surg.* 27 917–925. 10.1007/s11695-016-2399-2 27738970

[B111] Nguyen N. T. Varela J. E. (2017). Bariatric surgery for obesity and metabolic disorders: State of the art. *Nat. Rev. Gastroenterol. Hepatol.* 14 160–169. 27899816 10.1038/nrgastro.2016.170

[B112] Nickel F. Tapking C. Benner L. Sollors J. Billeter A. T. Kenngott H. G. (2018). Bariatric surgery as an efficient treatment for non-alcoholic fatty liver disease in a prospective study with 1-year follow-up: BariScan study. *Obes. Surg.* 28 1342–1350. 10.1007/s11695-017-3012-z 29119336

[B113] Nier A. Huber Y. Labenz C. Michel M. Bergheim I. Schattenberg J. M. (2020). Adipokines and endotoxemia correlate with hepatic steatosis in non-alcoholic fatty liver disease (NAFLD). *Nutrients* 12:699. 10.3390/nu12030699 32151020 PMC7146245

[B114] Nobili V. Alisi A. Mosca A. Della Corte C. Veraldi S. De Vito R. (2018). Hepatic farnesoid X receptor protein level and circulating fibroblast growth factor 19 concentration in children with NAFLD. *Liver Int.* 38 342–349. 10.1111/liv.13531 28746779

[B115] Ocaña-Wilhelmi L. Martín-Núñez G. M. Ruiz-Limón P. Alcaide J. García-Fuentes E. Gutiérrez-Repiso C. (2021). Gut microbiota metabolism of bile acids could contribute to the bariatric surgery improvements in extreme obesity. *Metabolites* 11:733. 10.3390/metabo11110733 34822391 PMC8620296

[B116] Park J. W. Kim H. Y. Kim M. G. Jeong S. Yun C. Han S. H. (2019). Short-chain fatty acids inhibit staphylococcal lipoprotein-induced nitric oxide production in murine macrophages. *Immune Netw.* 19:e9. 10.4110/in.2019.19.e9 31089436 PMC6494764

[B117] Park M. Y. Kim S. J. Ko E. K. Ahn S. H. Seo H. Sung M. K. (2016). Gut microbiota-associated bile acid deconjugation accelerates hepatic steatosis in ob/ob mice. *J. Appl. Microbiol.* 121 800–810. 10.1111/jam.13158 27111464

[B118] Parséus A. Sommer N. Sommer F. Caesar R. Molinaro A. Ståhlman M. (2017). Microbiota-induced obesity requires farnesoid X receptor. *Gut* 66 429–437. 10.1136/gutjnl-2015-310283 26740296 PMC5534765

[B119] Perdomo C. M. Gómez-Ambrosi J. Becerril S. Valentí V. Moncada R. Fernández-Sáez E. M. (2021). Role of ANGPTL8 in NAFLD improvement after bariatric surgery in experimental and human obesity. *Int. J. Mol. Sci.* 22:12945. 10.3390/ijms222312945 34884755 PMC8657645

[B120] Potthoff M. J. Boney-Montoya J. Choi M. He T. Sunny N. E. Satapati S. (2011). FGF15/19 regulates hepatic glucose metabolism by inhibiting the CREB-PGC-1α pathway. *Cell Metab.* 13 729–738. 10.1016/j.cmet.2011.03.019 21641554 PMC3131185

[B121] Puri P. Daita K. Joyce A. Mirshahi F. Santhekadur P. K. Cazanave S. (2017). The presence and severity of nonalcoholic steatohepatitis is associated with specific changes in circulating bile acids. *Hepatology* 67 534–548. 28696585 10.1002/hep.29359 PMC5764808

[B122] Qin J. Li Y. Cai Z. Li S. Zhu J. Zhang F. (2012). A metagenome-wide association study of gut microbiota in type 2 diabetes. *Nature* 490 55–60. 23023125 10.1038/nature11450

[B123] Rahman K. Desai C. Iyer S. S. Thorn N. E. Kumar P. Liu Y. (2016). Loss of junctional adhesion molecule a promotes severe steatohepatitis in mice on a diet high in saturated fat, fructose, and cholesterol. *Gastroenterology* 151 733–746.e12. 10.1053/j.gastro.2016.06.022 27342212 PMC5037035

[B124] Raman M. Ahmed I. Gillevet P. M. Probert C. S. Ratcliffe N. M. Smith S. (2013). Fecal microbiome and volatile organic compound metabolome in obese humans with nonalcoholic fatty liver disease. *Clin. Gastroenterol. Hepatol.* 11 868–875.e3. 23454028 10.1016/j.cgh.2013.02.015

[B125] Rao R. K. Seth A. Sheth P. (2004). Recent advances in alcoholic liver disease I. Role of intestinal permeability and endotoxemia in alcoholic liver disease. *Am. J. Physiol. Gastrointest. Liver Physiol.* 286 G881–G884. 10.1152/ajpgi.00006.2004 15132946

[B126] Rau M. Rehman A. Dittrich M. Groen A. K. Hermanns H. M. Seyfried F. (2018). Fecal SCFAs and SCFA-producing bacteria in gut microbiome of human NAFLD as a putative link to systemic T-cell activation and advanced disease. *United Eur. Gastroenterol. J.* 6 1496–1507. 10.1177/2050640618804444 30574320 PMC6297934

[B127] Ren N. Xing D. Rittmann B. E. Zhao L. Xie T. Zhao X. (2007). Microbial community structure of ethanol type fermentation in bio-hydrogen production. *Environ. Microbiol.* 9 1112–1125. 10.1111/j.1462-2920.2006.01234.x 17472628

[B128] Ridaura V. K. Faith J. J. Rey F. E. Cheng J. Duncan A. E. Kau A. L. (2013). Gut microbiota from twins discordant for obesity modulate metabolism in mice. *Science* 341:1241214. 10.1126/science.1241214 24009397 PMC3829625

[B129] Ridlon J. M. Kang D. J. Hylemon P. B. (2006). Bile salt biotransformations by human intestinal bacteria. *J. Lipid Res.* 47 241–259. 16299351 10.1194/jlr.R500013-JLR200

[B130] Risstad H. Kristinsson J. A. Fagerland M. W. le Roux C. W. Birkeland K. I. Gulseth H. L. (2017). Bile acid profiles over 5 years after gastric bypass and duodenal switch: Results from a randomized clinical trial. *Surg. Obes. Relat. Dis.* 13 1544–1553. 10.1016/j.soard.2017.05.024 28756050

[B131] Rivera C. A. Adegboyega P. van Rooijen N. Tagalicud A. Allman M. Wallace M. (2007). Toll-like receptor-4 signaling and Kupffer cells play pivotal roles in the pathogenesis of non-alcoholic steatohepatitis. *J. Hepatol.* 47 571–579. 17644211 10.1016/j.jhep.2007.04.019 PMC2094119

[B132] Romero-Gómez M. Zelber-Sagi S. Trenell M. (2017). Treatment of NAFLD with diet, physical activity and exercise. *J. Hepatol.* 67 829–846. 28545937 10.1016/j.jhep.2017.05.016

[B133] Rossell J. Brindefalk B. Baena-Fustegueras J. A. Peinado-Onsurbe J. Udekwu K. I. (2020). Diet change affects intestinal microbiota restoration and improves vertical sleeve gastrectomy outcome in diet-induced obese rats. *Eur. J. Nutr.* 59 3555–3564. 10.1007/s00394-020-02190-8 32055963 PMC7669806

[B134] Safari Z. Gérard P. (2019). The links between the gut microbiome and non-alcoholic fatty liver disease (NAFLD). *Cell. Mol. Life Sci.* 76 1541–1558. 10.1007/s00018-019-03011-w 30683985 PMC11105223

[B135] Samuel B. S. Shaito A. Motoike T. Rey F. E. Backhed F. Manchester J. K. (2008). Effects of the gut microbiota on host adiposity are modulated by the short-chain fatty-acid binding G protein-coupled receptor, Gpr41. *Proc. Natl. Acad. Sci. U.S.A.* 105 16767–16772. 10.1073/pnas.0808567105 18931303 PMC2569967

[B136] Sánchez-Alcoholado L. Gutiérrez-Repiso C. Gómez-Pérez A. M. García-Fuentes E. Tinahones F. J. Moreno-Indias I. (2019). Gut microbiota adaptation after weight loss by Roux-en-Y gastric bypass or sleeve gastrectomy bariatric surgeries. *Surg. Obes. Relat. Dis.* 15 1888–1895. 31648978 10.1016/j.soard.2019.08.551

[B137] Sanchez-Carrillo S. Ciordia S. Rojo D. Zubeldia-Varela E. Méndez-García C. Martínez-Martínez M. (2021). A body weight loss- and health-promoting gut microbiota is established after bariatric surgery in individuals with severe obesity. *J. Pharm. Biomed. Anal.* 193:113747. 10.1016/j.jpba.2020.113747 33217711

[B138] Santos A. A. Afonso M. B. Ramiro R. S. Pires D. Pimentel M. Castro R. E. (2020). Host miRNA-21 promotes liver dysfunction by targeting small intestinal *Lactobacillus* in mice. *Gut Microbes* 12:1840766. 10.1080/19490976.2020.1840766 33300439 PMC7733982

[B139] Sarkola T. Eriksson C. J. (2001). Effect of 4-methylpyrazole on endogenous plasma ethanol and methanol levels in humans. *Alcohol. Clin. Exp. Res.* 25 513–516. 11329490

[B140] Scheithauer T. P. M. Davids M. Winkelmeijer M. Verdoes X. Aydin Ö. de Brauw M. (2022). Compensatory intestinal antibody response against pro-inflammatory microbiota after bariatric surgery. *Gut Microbes* 14:2031696. 10.1080/19490976.2022.2031696 35130127 PMC8824225

[B141] Sender R. Fuchs S. Milo R. (2016). Are we really vastly outnumbered? Revisiting the ratio of bacterial to host cells in humans. *Cell* 164 337–340. 10.1016/j.cell.2016.01.013 26824647

[B142] Shen N. Caixàs A. Ahlers M. Patel K. Gao Z. Dutia R. (2019). Longitudinal changes of microbiome composition and microbial metabolomics after surgical weight loss in individuals with obesity. *Surg. Obes. Relat. Dis.* 15 1367–1373. 10.1016/j.soard.2019.05.038 31296445 PMC6722012

[B143] Shin J. Jung S. Kim S. Kang M. Kim M. Joung H. (2019). Differential effects of typical Korean versus American-style diets on gut microbial composition and metabolic profile in healthy overweight Koreans: A randomized crossover trial. *Nutrients* 11:2450. 10.3390/nu11102450 31615057 PMC6835328

[B144] Sorbara M. T. Pamer E. G. (2022). Microbiome-based therapeutics. *Nat. Rev. Microbiol.* 20 365–380. 34992261 10.1038/s41579-021-00667-9

[B145] Steinert R. E. Peterli R. Keller S. Meyer-Gerspach A. C. Drewe J. Peters T. (2013). Bile acids and gut peptide secretion after bariatric surgery: A 1-year prospective randomized pilot trial. *Obesity* 21 E660–E668. 10.1002/oby.20522 23804517

[B146] Steinert R. E. Rehman A. Souto Lima E. J. Agamennone V. Schuren F. H. J. Gero D. (2020). Roux-en-Y gastric bypass surgery changes fungal and bacterial microbiota in morbidly obese patients—a pilot study. *PLoS One* 15:e0236936. 10.1371/journal.pone.0236936 32735609 PMC7394366

[B147] Stienstra R. Saudale F. Duval C. Keshtkar S. Groener J. E. M. van Rooijen N. (2010). Kupffer cells promote hepatic steatosis via interleukin-1β-dependent suppression of peroxisome proliferator-activated receptor α activity. *Hepatology* 51 511–522. 10.1002/hep.23337 20054868

[B148] Sun L. Pang Y. Wang X. Wu Q. Liu H. Liu B. (2019). Ablation of gut microbiota alleviates obesity-induced hepatic steatosis and glucose intolerance by modulating bile acid metabolism in hamsters. *Acta Pharm. Sin. B* 9 702–710. 10.1016/j.apsb.2019.02.004 31384531 PMC6664038

[B149] Sung J. J. Chiu P. W. Chan F. K. L. Lau J. Y. Goh K. Ho L. H. (2018). Asia-Pacific working group consensus on non-variceal upper gastrointestinal bleeding: An update 2018. *Gut* 67 1757–1768. 10.1136/gutjnl-2018-316276 29691276 PMC6145289

[B150] Svegliati-Baroni G. Saccomanno S. Rychlicki C. Agostinelli L. De Minicis S. Candelaresi C. (2011). Glucagon-like peptide-1 receptor activation stimulates hepatic lipid oxidation and restores hepatic signalling alteration induced by a high-fat diet in nonalcoholic steatohepatitis. *Liver Int.* 31 1285–1297. 10.1111/j.1478-3231.2011.02462.x 21745271

[B151] Syn N. L. Cummings D. E. Wang L. Z. Lin D. J. Zhao J. J. Loh M. (2021). Association of metabolic-bariatric surgery with long-term survival in adults with and without diabetes: A one-stage meta-analysis of matched cohort and prospective controlled studies with 174?772 participants. *Lancet* 397 1830–1841. 33965067 10.1016/S0140-6736(21)00591-2

[B152] Talavera-Urquijo E. Beisani M. Balibrea J. M. Alverdy J. C. (2020). Is bariatric surgery resolving NAFLD via microbiota-mediated bile acid ratio reversal? A comprehensive review. *Surg. Obes. Relat. Dis.* 16 1361–1369. 10.1016/j.soard.2020.03.013 32336663

[B153] Tan X. Liu Y. Long J. Chen S. Liao G. Wu S. (2019). Trimethylamine-N-oxide aggravates liver steatosis through modulation of bile acid metabolism and inhibition of farnesoid X receptor signaling in nonalcoholic fatty liver disease. *Mol. Nutr. Food Res.* 63:1900257. 10.1002/mnfr.201900257 31095863

[B154] Tarao K. So K. Moroi T. Ikeuchi T. Suyama T. (1977). Detection of endotoxin in plasma and ascitic fluid of patients with cirrhosis: Its clinical significance. *Gastroenterology* 73 539–542. 10.1016/S0016-5085(19)32137-7 892353

[B155] Thaiss C. A. Levy M. Grosheva I. Zheng D. Soffer E. Blacher E. (2018). Hyperglycemia drives intestinal barrier dysfunction and risk for enteric infection. *Science* 359 1376–1383. 10.1126/science.aar3318 29519916

[B156] Tremaroli V. Karlsson F. Werling M. Ståhlman M. Kovatcheva-Datchary P. Olbers T. (2015). Roux-en-Y gastric bypass and vertical banded gastroplasty induce long-term changes on the human gut microbiome contributing to fat mass regulation. *Cell Metab.* 22 228–238. 10.1016/j.cmet.2015.07.009 26244932 PMC4537510

[B157] Triger D. R. Boyer T. D. Levin J. (1978). Portal and systemic bacteraemia and endotoxaemia in liver disease. *Gut* 19 935–939. 10.1136/gut.19.10.935 710964 PMC1412358

[B158] Turnbaugh P. J. Ley R. E. Mahowald M. A. Magrini V. Mardis E. R. Gordon J. I. (2006). An obesity-associated gut microbiome with increased capacity for energy harvest. *Nature* 444 1027–1031. 10.1038/nature05414 17183312

[B159] Turner J. R. (2009). Intestinal mucosal barrier function in health and disease. *Nat. Rev. Immunol.* 9 799–809. 19855405 10.1038/nri2653

[B160] Wang B. Jiang X. Cao M. Ge J. Bao Q. Tang L. (2016). Altered fecal microbiota correlates with liver biochemistry in nonobese patients with non-alcoholic fatty liver disease. *Sci. Rep.* 6:32002. 10.1038/srep32002 27550547 PMC4994089

[B161] Wang Z. Klipfell E. Bennett B. J. Koeth R. Levison B. S. DuGar B. (2011). Gut flora metabolism of phosphatidylcholine promotes cardiovascular disease. *Nature* 472 57–63. 10.1038/nature09922 21475195 PMC3086762

[B162] Watanabe M. Horai Y. Houten S. M. Morimoto K. Sugizaki T. Arita E. (2011). Lowering bile acid pool size with a synthetic farnesoid X receptor (FXR) agonist induces obesity and diabetes through reduced energy expenditure. *J. Biol. Chem.* 286 26913–26920. 10.1074/jbc.M111.248203 21632533 PMC3143650

[B163] Watanabe M. Houten S. M. Wang L. Moschetta A. Mangelsdorf D. J. Heyman R. A. (2004). Bile acids lower triglyceride levels via a pathway involving FXR, SHP, and SREBP-1c. *J. Clin. Investig.* 113 1408–1418. 10.1172/JCI21025 15146238 PMC406532

[B164] Wirth K. M. Sheka A. C. Kizy S. Irey R. Benner A. Sieger G. (2020). Bariatric surgery is associated with decreased progression of nonalcoholic fatty liver disease to cirrhosis: A retrospective cohort analysis. *Ann. Surg.* 272 32–39. 10.1097/SLA.0000000000003871 32224733 PMC12243115

[B165] Wu J. Wang K. Wang X. Pang Y. Jiang C. (2021). The role of the gut microbiome and its metabolites in metabolic diseases. *Protein Cell* 12 360–373. 33346905 10.1007/s13238-020-00814-7 PMC8106557

[B166] Yang G. Lee H. E. Lee J. Y. (2016). A pharmacological inhibitor of NLRP3 inflammasome prevents non-alcoholic fatty liver disease in a mouse model induced by high fat diet. *Sci. Rep.* 6:24399. 27075683 10.1038/srep24399 PMC4830938

[B167] Yao Z. Vance D. E. (1990). Reduction in VLDL, but not HDL, in plasma of rats deficient in choline. *Biochem. Cell Biol.* 68 552–558. 10.1139/o90-079 2344402

[B168] Yeo S. C. Ong W. M. Cheng K. Tan C. H. (2019). Weight loss after bariatric surgery predicts an improvement in the non-alcoholic fatty liver disease (NAFLD) fibrosis score. *Obes. Surg.* 29 1295–1300. 30635812 10.1007/s11695-018-03676-5

[B169] Younossi Z. Anstee Q. M. Marietti M. Hardy T. Henry L. Eslam M. (2018). Global burden of NAFLD and NASH: Trends, predictions, risk factors and prevention. *Nat. Rev. Gastroenterol. Hepatol.* 15 11–20. 10.1038/nrgastro.2017.109 28930295

[B170] Younossi Z. M. Blissett D. Blissett R. Henry L. Stepanova M. Younossi Y. (2016). The economic and clinical burden of nonalcoholic fatty liver disease in the United States and Europe. *Hepatology* 64 1577–1586. 10.1002/hep.28785 27543837

[B171] Younossi Z. M. Corey K. E. Lim J. K. (2021). AGA clinical practice update on lifestyle modification using diet and exercise to achieve weight loss in the management of nonalcoholic fatty liver disease: Expert review. *Gastroenterology* 160 912–918. 10.1053/j.gastro.2020.11.051 33307021

[B172] Yousseif A. Emmanuel J. Karra E. Millet Q. Elkalaawy M. Jenkinson A. D. (2014). Differential effects of laparoscopic sleeve gastrectomy and laparoscopic gastric bypass on appetite, circulating acyl-ghrelin, peptide YY3-36 and active GLP-1 levels in non-diabetic humans. *Obes. Surg.* 24 241–252. 10.1007/s11695-013-1066-0 23996294 PMC3890046

[B173] Yun Y. Kim H. Lee E. Ryu S. Chang Y. Shin H. (2019). Fecal and blood microbiota profiles and presence of nonalcoholic fatty liver disease in obese versus lean subjects. *PLoS One* 14:e0213692. 10.1371/journal.pone.0213692 30870486 PMC6417675

[B174] Zhao J. Liu P. Wu Y. Guo P. Liu L. Ma N. (2018). Dietary fiber increases butyrate-producing bacteria and improves the growth performance of weaned piglets. *J. Agric. Food Chem.* 66 7995–8004. 10.1021/acs.jafc.8b02545 29986139

[B175] Zhou D. Pan Q. Xin F. Zhang R. He C. Chen G. (2017). Sodium butyrate attenuates high-fat diet-induced steatohepatitis in mice by improving gut microbiota and gastrointestinal barrier. *World J. Gastroenterol.* 23 60–75. 10.3748/wjg.v23.i1.60 28104981 PMC5221287

[B176] Zhu L. Baker S. S. Gill C. Liu W. Alkhouri R. Baker R. D. (2013). Characterization of gut microbiomes in nonalcoholic steatohepatitis (NASH) patients: A connection between endogenous alcohol and NASH. *Hepatology* 57 601–609. 10.1002/hep.26093 23055155

[B177] Zhu R. Baker S. S. Moylan C. A. Abdelmalek M. F. Guy C. D. Zamboni F. (2016). Systematic transcriptome analysis reveals elevated expression of alcohol-metabolizing genes in NAFLD livers. *J. Pathol.* 238 531–542. 10.1002/path.4650 26415102

